# Mapping Small-World Properties through Development in the Human Brain: Disruption in Schizophrenia

**DOI:** 10.1371/journal.pone.0096176

**Published:** 2014-04-30

**Authors:** Dardo Tomasi, Nora D. Volkow

**Affiliations:** 1 National Institute on Alcohol Abuse and Alcoholism, Bethesda, Maryland, United States of America; 2 National Institute on Drug Abuse, Bethesda, Maryland, United States of America; Beijing Normal University, China

## Abstract

Evidence from imaging studies suggests that the human brain has a small-world network topology that might be disrupted in certain brain disorders. However, current methodology is based on global graph theory measures, such as clustering, *C*, characteristic path length, *L*, and small-worldness, *S*, that lack spatial specificity and are insufficient to identify regional brain abnormalities. Here we propose novel ultra-fast methodology for mapping local properties of brain network topology such as local *C*, *L* and *S* (*lC*, *lL* and *lS*) in the human brain at 3-mm isotropic resolution from ‘resting-state’ magnetic resonance imaging data. Test-retest datasets from 40 healthy children/adolescents were used to demonstrate the overall good reliability of the measures across sessions and computational parameters (intraclass correlation > 0.5 for *lC* and *lL*) and their low variability across subjects (< 29%). Whereas regions with high local functional connectivity density (*l*FCD; local degree) in posterior parietal and occipital cortices demonstrated high *lC* and short *lL*, subcortical regions (globus pallidus, thalamus, hippocampus and amygdala), cerebellum (lobes and vermis), cingulum and temporal cortex also had high, *lS*, demonstrating stronger small-world topology than other hubs. Children/adolescents had stronger *l*FCD, higher *lC* and longer *lL* in most cortical regions and thalamus than 74 healthy adults, consistent with pruning of functional connectivity during maturation. In contrast, *l*FCD, *lC* and *lL* were weaker in thalamus and midbrain, and *lL* was shorter in frontal cortical regions and cerebellum for 69 schizophrenia patients than for 74 healthy controls, suggesting exaggerated pruning of connectivity in schizophrenia. Follow up correlation analyses for seeds in thalamus and midbrain uncovered lower positive connectivity of these regions in thalamus, putamen, cerebellum and frontal cortex (cingulum, orbitofrontal, inferior frontal) and lower negative connectivity in auditory, visual, motor, premotor and somatosensory cortices for schizophrenia patients than for controls, consistent with prior findings of thalamic disconnection in schizophrenia.

## Introduction

Graph theory postulates that real networks have a ‘small-world’ topology with high clustering and short path length that ensures within-network maximal communication rate with minimal wiring cost [1]. The human brain is a complex network of highly interconnected regions and an example of a real network with small-world topology [2]. Its complex organization can be characterized by two meaningful global parameters, clustering and path length [3]–[6], that can reveal global functional and/or structural abnormalities in the human brain [7]. However, global brain parameters lack regional specificity and might have limited impact for the identification of dysfunctional brain regions and for monitoring therapeutic responses in specific networks. Here we propose novel voxelwise methodology for mapping local small-world network properties in the human brain.

Consistent with the small-world network topology [1], the architecture of the brain include few “hubs” (nodes with high connectivity degree) interconnecting distributed local networks, and abundant weakly connected nodes [7]. Brain functional connectivity computed from magnetic resonance imaging (MRI) data collected in resting conditions demonstrated the distribution of functional connectivity hubs in the human brain [8]–[11], and is a powerful method for studying the topology of the brain in health and disease conditions [10], [12], [13] with high spatial resolution (3-mm isotropic). However, the lack of additional voxelwise graph theory measures (clustering and path length) limits our interpretation of abnormalities in the strength of the connectivity hubs in psychiatric populations.

Schizophrenia is characterized by abnormal structural and functional connectivity between distributed brain networks, which results in disconnection of neuronal processing [14]–[16]. Functional connectivity studies in 12 schizophrenia patients that assessed network topology from 72 anatomical regions-of-interest in the brain reported reduced clustering and small-worldness, a measure that gauges the strength of the small-world characteristics of a network [17], in schizophrenia [18]. Similar results were reported for 24 schizophrenia patients in studies of brain topology that used 75 functional regions-of-interest identified by independent component analysis [19]. We hypothesized that voxelwise functional connectivity studies at 3-mm spatial resolution in schizophrenia patients would reveal abnormal degree, clustering and characteristic path length in specific brain regions.

Here, we propose a voxelwise approach to map graph theory properties in the brain. Specifically, we show that functional connectivity density (FCD) mapping [11] can be extended to map the characteristic path length, clustering and small-worldness of the local networks functionally connected to each brain voxel. Since the default mode network (DMN) and visual cortex are highly interconnected with other brain regions and include the most prominent connectivity hubs in the brain [20] we hypothesized uneven spatial distributions with local maxima in DMN and in the visual cortex for the graph theory metrics. We assessed the reliability of the graph theory metrics across subjects, sessions and parameters using intraclass correlation (ICC) and test-retest MRI datasets from 40 healthy children/adolescents. Since functional connectivity can detect brain maturation changes from childhood to adulthood [21], we tested the sensitivity of the graph theory measures to brain development with the hypothesis that children/adolescents would show lower local FCD (*l*FCD), clustering (*lC*), characteristic path length (*lL*) and small-worldness (*lS*) than adults. To test the sensitivity of the method to brain pathology we assessed differences in *l*FCD, *lC*, *lL* and *lS* between schizophrenia patients and healthy control subjects using statistical parametric mapping. Based on prior reports of disrupted brain organization in schizophrenia [22], [23] we hypothesized that patients would show lower *l*FCD and *lC* in subcortical and prefrontal brain regions than controls.

## Methods

### Ethics Statement

This study is based on existing datasets that were distributed with the approval of the corresponding Institutional Review Boards (IRB) and in compliance with the Health Insurance Portability and Accountability (HIPAA) privacy rules via the International Neuroimaging Data-sharing Initiative (http://fcon_1000.projects.nitrc.org/). The authors did not have any role in the collection of the data nor had access to any identifying information from the participants. The test-retest study included 40 children from the WashU research site of the ADHD-200 dataset (http://fcon_1000.projects.nitrc.org/indi/adhd200/), which gave assent with parental written informed consent in accordance with the guidelines and approval of the Washington University Human Studies Committee. The schizophrenia study included 143 adult subjects from the COBRE dataset (http://fcon_1000.projects.nitrc.org/indi/retro/cobre.html), which gave written informed consent in accordance with the Human Subjects Research Review Committee (HRRC) of the University of New Mexico Health Sciences Center.

### Subjects

The test-retest studies included 40 healthy young subjects (age: 13± 4 years; 17 females), each contributing with 3 resting state scans that were collected in a 3T Siemens Magnetom Trio MRI scanner while the subjects kept their eyes open using a single-shot gradient echo planar (EPI) imaging pulse sequence with 64 phase encodings, 132 time points, 4-mm isotropic resolution, 2500 ms repetition time (TR), 27 ms echo time (TE) and 32 axial slices covering the whole brain. All subjects were recruited and screened at St. Louis Children's Hospital, Washington University in Saint Louis.

The schizophrenia study included 69 patients (age: 38 ± 14 years; 14 females) and 74 (age: 36 ± 12 years; 23 females) control adult subjects. The corresponding MRI time series had 150 time points and were collected in a 3T Siemens Magnetom Trio MRI scanner using single-shot gradient EPI (TE/TR  =  29/2000ms; 3.75 × 3.75 × 4 mm^3^ resolution; 32 slices). All subjects were recruited and screened at the University of New Mexico by personnel of the Mind Research Network. Subjects were excluded if they had; history of neurological disorder, history of mental retardation, history of severe head trauma with more than 5 minutes loss of consciousness, history of substance abuse or dependence within the last 12 months. Diagnostic information was collected using the Structured Clinical Interview for DSM Disorders (SCID). Patients were diagnosed as paranoid (40), residual (12), schizoaffective (5), unspecified (8), and with co-morbidity [24], [25] [bipolar I disorder (1) and senile delirium (1)]. Unfortunately the database did not contain information regarding the medication status of the schizophrenia patients.

### Image preprocessing

The statistical parametric mapping package SPM8 (Wellcome Trust Centre for Neuroimaging, London, UK) was used for image realignment and spatial normalization to the stereotactic space of the Montreal Neurological Institute (MNI). For this purpose a 12-parameters affine transformation with medium regularization, 16-nonlinear iterations, voxel size of 3 × 3 × 3 mm^3^ and the EPI.mnc template included in the SPM package were used. Subsequent preprocessing steps were carried out using IDL (ITT Visual Information Solutions, Boulder, CO).

Time points that were severely contaminated with motion were removed using a “scrubbing” method [26]. Specifically, we computed the root mean square variance across voxels (DVARS) of the differences in % BOLD intensity, *I*_i_, between adjacent time points, 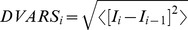, where the brackets denote averaging over imaging voxels. For every time point, *i*, we also computed framewise displacements, 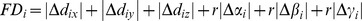, from head translations (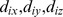) and rotations (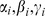), the six image realignment parameters from SPM. A radius *r*  =  50 mm, approximately the mean distance from the center of the MNI space to the cortex, was used to convert angle rotations to displacements. The average head displacement across time points was significantly higher for adults than for children and for schizophrenia patients than for controls (<FD>  =  0.26 ± 0.16 for patients, 0.17 ± 0.10 for controls and 0.07 ± 0.04 for children; P < 0.0002). Image time points with *FD*_i_ > 0.5 mm and *DVARS*_i_ > 0.5% were considered potentially contaminated with motion artifacts and excluded from the time series [26]. The scrubbing algorithm excluded a maximum of 5 time points per time series (< 3% of the time points).

The global signal intensity was normalized across time points and the time-varying realignment parameters (3 translations and 3 rotations) were used in a multilinear regression approach to minimize motion related fluctuations in the MRI signals [11]. Magnetic field drifts of the scanner and physiologic noise of high frequency components were minimized using 0.01–0.10 Hz band-pass temporal filtering [11]. Voxels with poor signal-to-noise as a function of time (SNR_t_ < 50) were eliminated to minimize voxels with excessive susceptibility-related signal-loss artifacts. These preprocessed MRI time series were saved in hard drive for subsequent analyses.

### Local degree

The strength of the functional connectivity, 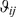, between voxels *i* and *j* in the brain, was assessed with Pearson correlations, and a correlation threshold of *R*_T1_  =  0.40 was used to compute binary coefficients, 
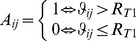
. In this study, the selected correlation threshold ensures significant correlations between time-varying signal fluctuations at P < 0.0005. Two additional values, *R*_T1_  =  0.45 and 0.50, were used to assess the robustness of the results as a function of different threshold criteria. The local functional connectivity density (*l*FCD), which reflects the number of neighbors in the local functional connectivity cluster, 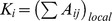, was computed in IDL using a 'growing' algorithm [11]. Specifically, a voxel (*x*_j_) was added to the list of voxels functionally connected with x_i_ only if it was adjacent to a voxel that was linked to *x*_i_ by a continuous path of functionally connected voxels and 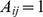. This calculation was repeated for all voxels that were adjacent to voxels that belong to the list of voxels functionally connected to *x*_i_ in an iterative manner until no new voxel could be added to the list. Then the calculation was initiated for a different *x*_i_.

### Local clustering and path length

Clustering is a property of densely interconnected groups of voxels that reflects their functional segregation, i.e. their ability to perform specialized neural processing in the brain [27]. A second correlation threshold, *R*_T2_  =  0.65 > *R*_T1_, was used to construct the binary undirected connectivity matrix, 
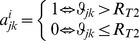
, which was used to estimate the clustering, path length and small-worldness of the local network functionally connected to voxel *i*. Two additional values, *R*_T2_  =  0.70 and 0.75, were used to assess the robustness of these measures as a function of different threshold criteria. Note that the (K_i_ × K_i_) ***a*** *^i^* adjacency matrix has *m* edges 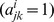 representing significant interactions 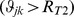 between nodes in the local network of voxel *i*.

The number of “triangles” (i.e. sets of three neighbor nodes interconnected by edges) in the vicinity of *j*-node is a measure of the segregation of the network [27]. The number of triangles within the network of voxel *i* was computed using the adjacency matrix according to [27],



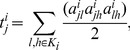
(1)from which the clustering coefficient for the local network functionally connected to node *i*, *lC*(*i*), was computed in terms of the degree (number of edges), 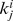, of each node in the local network in analogy with the definition of clustering [1],



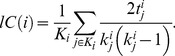
(2)


This measure of segregation quantify the presence of clusters/modules surrounding each voxel within the local network functionally connected to voxel *i*.

The shortest path length or distance between two nodes (*l* and *j*) of the local network of voxel *i* was computed using the adjacency matrix as the minimum number of edges that must be traversed to get from one node to the other [27],



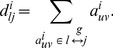
(3)Where 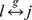 is the shortest geodesic path between nodes *l* and *j*. The characteristic path length of the *local* network functionally connected to voxel *i*, *lL*(*i*), was computed as the mean value of the shortest path length over all node pairs in the local network in analogy with the definition of characteristic path length [1],



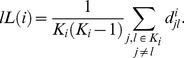
(4)


Note that *lL^i^* measures of the functional integration of the local networks, which is predominantly influenced by long paths.

### Local small-worldness

Formally, a real network is considered to be a small-world network if it has a similar path length but greater clustering than an equivalent random graph, constructed with a uniform probability and the same numbers of nodes, K, and edges, m, as the real network. We computed the local small-worldness in analogy to the definition small-worldness [17],



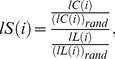
(5)of the *local* networks functionally connected to the brain voxels to assess the small-world topology of the brain regions. Comparable random graphs were constructed with the same K_i_ and m_i_ as the local network functionally connected to voxel *i*, using an IDL uniform random number generator with Bays-Durham shuffling [28]. Note that we mapped small-world properties in the brain by computing *lL*, *lC* and *lS* for a total of 57,713 local networks (one network per voxel in the gray matter) for each of the resting state MRI scans in this study. The average computation time required for the calculation of these graph theory metrics was 15 ± 5 minutes per subject in a dual Xeon (X5680; 3.33GHz; 24 threads) personal computer with 28 GB of random access memory running 64-bit Windows 7.

### Intraclass correlation

The two-way mixed single measures intraclass correlation coefficient (ICC) was used to assess the reliability of the *l*FCD, *lC*, *lL* and *lS* maps. Specifically, the consistency of these graph theory measures across all different raters (correlation thresholds and resting-state sessions) was computed in terms of between-subjects mean squares (BMS), residuals mean squares (EMS) and the number of raters,



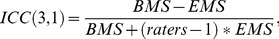
(6)using the IPN toolbox for test-retest reliability analysis of imaging datasets (http://www.mathworks.com/matlabcentral/fileexchange/22122-ipn-tools-for-test-retest-reliability-analysis). Independent ICC analyses were carried for each of the graph theory measures, which included maps corresponding to all subjects and sessions in the WashU dataset as well as maps computed with different *R*_T1_ and *R*_T2_ thresholds. Note that the ICC(3,1) voxel values range from 0 (no reliability) to 1 (perfect reliability across sessions and thresholds) and that ICC(3,1) > 0.5 indicate significant reliability in this study. The (*R*_T1_, *R*_T2_) set that maximized the whole-brain mean ICC(3,1) was used for subsequent analyses.

### Seed-voxel correlations

Standard seed-voxel correlation analyses were used to compute the functional connectivity (FC) for a bilateral thalamic seed (x  =  ±6 mm, y  =  −3 mm, z  =  0 mm; 54 voxels) and for a bilateral seed in the substantia nigra (SN) of midbrain (x  =  ±12 mm, y  =  −15 mm, z  =  −12 mm; 54 voxels), regions that demonstrated lower *lC*, shorter *lL* and weaker *l*FCD in schizophrenic patients compared to controls. These patterns were used to identify the patients' dysfunctional thalamocortical, mesocortical and nigrostriatal connectivity pathways.

### Statistical Analyses

The maps (*l*FCD, *lC*, *lL*, *lS* and FC) were spatially smoothed (8-mm) to minimize the variability of brain anatomy across subjects. As in our previous studies [11], grand mean scaling was used to control for differences in pulse sequence parameters and scanner between the WashU dataset and the COBRE dataset, allowing us to merge datasets from different research sites. Specifically, the mean value of the connectivity metrics across voxels in the brain and subjects was computed independently for the WashU dataset and the COBRE dataset as well as for *l*FCD, *lC*, *lL* and *lS*. These values were used as scaling factors for the corresponding individual maps. Principal component analysis (PCA), using the covariance of the imaging data without mean centering, was performed in IDL to analyze the variability of the graph theory measures across subjects.

Like Pearson correlation factors, which are distributed in the [−1, 1] interval, the *l*C coefficients are not suitable for statistical purposes because they do not have normal distribution in the interval (−∞, ∞). Specifically, *l*C coefficients lie in the interval [0, 1], and must be converted to standard scores, z, into the interval (−∞, ∞) for statistical purposes. Correlation factors were converted into standard scores using the Fisher transformation,



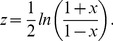
(7)For *lC* coefficients, a Fisher-like transformation was obtained by substituting *x*  =  2 *lC* – 1 in Eq. [7],



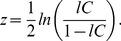
(8)This transformation was used to convert *lC* coefficients into standard scores for statistical purposes. One-way ANOVA with two zero-mean covariates (<FD> and gender) controlling for the confounding effects of micro movement [29] and gender [30] was used to assess differences in *l*FCD, *lC*, *lL* and *lS* between 40 healthy children from the WashU dataset (resting-state session 1) and 74 healthy adults from the COBRE dataset. Similarly, one-way ANOVA SPM8 model with two groups (74 healthy controls and 69 schizophrenia patients from the COBRE dataset) and three zero-mean covariates controlling for the confounding effects of micro movement, age [31] and gender was used to assess effects of schizophrenia, independently for *l*FCD, *lC*, *lL,* and *lS* as well as for thalamic and midbrain FC. For all voxelwise analyzes, statistical significance was set as P_FWE_ < 0.05, corrected for multiple comparisons at the cluster level with the random field theory and a family-wise error correction using a cluster-forming threshold P < 0.0005 and a minimum cluster size of 10 voxels.

### Region-of-interest (ROI) analyses

The population-average landmark- and surface-based atlas of the cerebral cortex [32] and the Automated Anatomical Labeling (AAL) atlas [33] provided in the installation package of the MRIcron image viewer (http://www.nitrc.org/projects/mricron) were used to identify cortical Brodmann areas (BA) and subcortical regions (thalamus, caudate, putamen, globus pallidus, hippocampus, parahippocampus, and amygdala, cerebellar lobes and cerebellar vermis in the MNI stereotactic space. Average values of *lC*, *lL*, *lS* and *l*FCD and their standard errors were computed within these ROIs using a custom IDL program.

## Results

### Clustering and characteristic path length

**Figure 1:**
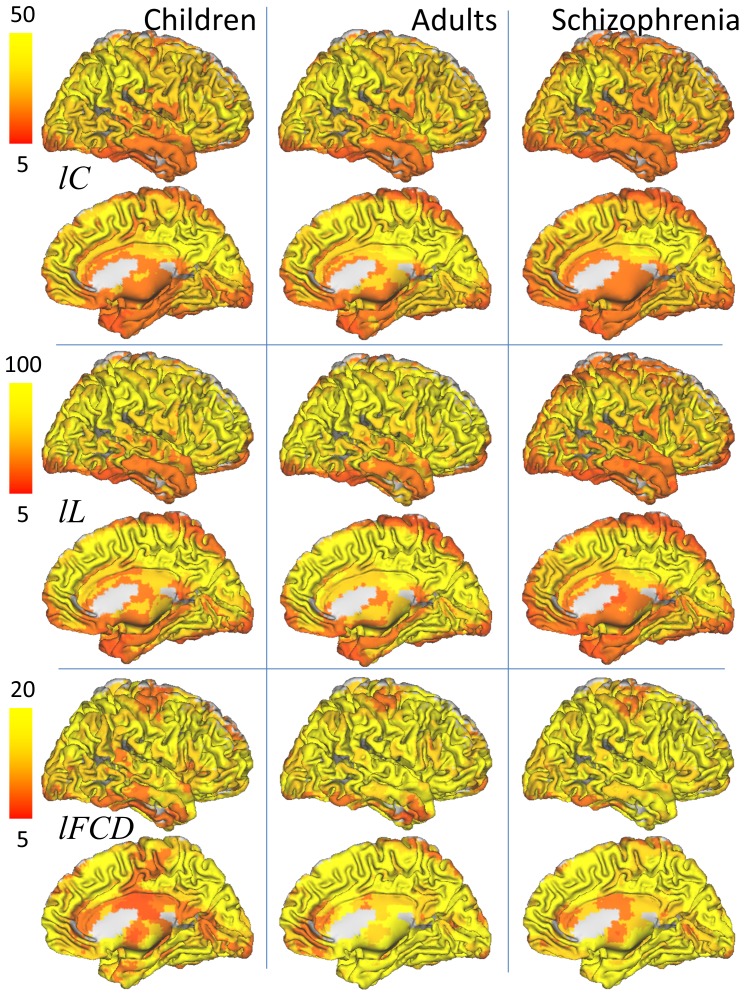
Statistical significance of graph theory metrics. T-score maps corresponding to local clustering, *lC*, characteristic path length, *lL*, and functional connectivity density (degree), *l*FCD, for 40 typically developing children, 74 healthy adults and 69 schizophrenia patients, superimposed on lateral and medial views of the human brain surface. Correlation thresholds: *R*_T1_  =  0. 50; *R*_T2_  =  0.65.

**Figure 2:**
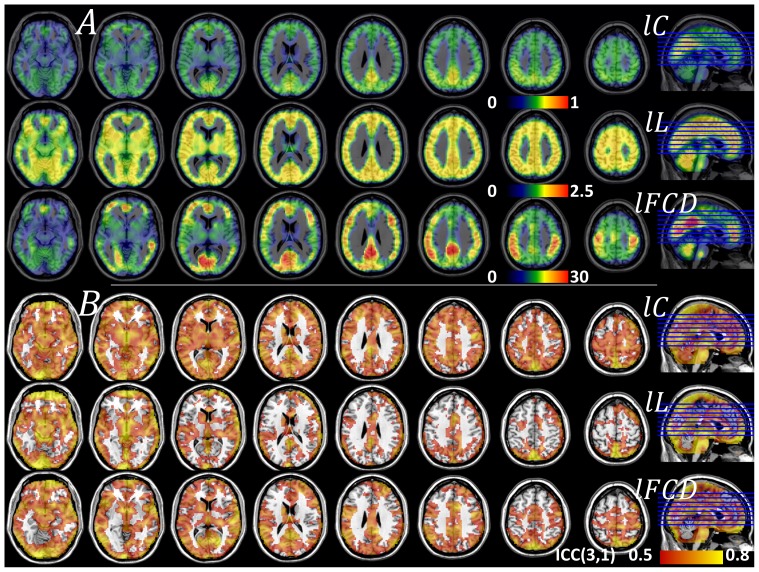
Regional measures of absolute graph theory properties. (A) Average strength of local clustering, *lC*, characteristic path length, *lL*, and functional connectivity density (degree), *l*FCD, of the networks functionally connected to each imaging voxel, superimposed on axial views of the human brain. (B) Maps of two-way mixed single measures intraclass correlation, ICC(3,1) demonstrating the good reliability (ICC > 0.5) of *lC*, *lL* and *l*FCD across sessions and method thresholds. Sample: 3 resting-state sessions from 40 healthy children (WashU dataset), correlation thresholds: *R*_T1_  =  0.40, 0.45 and 0.50; *R*_T2_  =  0.65, 0.70 and 0.75.

The average distributions of *lC*, *lL* and *l*FCD were highly significant for all brain regions (t-score > 5; Fig 1 and Table 1). In children/adolescents *l*C and *lL* were maximal in the visual cortex (BAs 17 and 19), posterior and anterior cingulum (BAs 23 and 10), superior and inferior parietal (BAs 2, 3 and 39) and frontal (BAs 45 and 47) cortices (Fig 2A and Table 1). The average whole-brain values of the local connectivity measures were: <*lC*>  =  0.35 ± 0.12 and <*lL*>  =  1.25 ± 0.40 and the average volume of the local networks was <*l*FCD>  =  11.9 ± 5.7 voxels. PCA demonstrated low variability of the *lC* and *lL* patterns across children/adolescents. Specifically, the principal component, which reflects the mean patterns (the imaging data did not have zero empirical mean), did not account for a small fraction of the variance of the *lC* (13%), *lL* (6%) and *l*FCD (31%) patterns across subjects (Figure S1).

**Table 1:** Strength, statistical significance and reliability for local measures of degree, clustering (*lC*) and characteristic path length (*lL*) at the locations of the functional connectivity density (*l*FCD) hubs in the brain of children/adolescents.

Region	BA	MNI coordinates [mm]			Local degree			Clustering			Path Length		
		x	y	z	lFCD (voxels)	T	ICC	*lC*	T	ICC	*lL*	T	ICC
**Posterior Cingulum**	23	−6	−48	33	43	20	0.63	0.61	81	0.63	1.80	108	0.56
**Calcarine**	17	−12	−72	12	34	15	0.71	0.55	48	0.72	1.70	109	0.48
**Inferior Parietal**	39	−51	−57	39	28	19	0.69	0.54	65	0.62	1.74	133	0.46
**Inferior Frontal**	45	45	21	24	27	24	0.46	0.51	58	0.56	1.79	147	0.34
**Postcentral**	3	42	−27	54	27	15	0.60	0.50	44	0.54	1.71	119	0.36
**Postcentral**	4	−42	−21	57	25	15	0.48	0.48	42	0.56	1.66	111	0.32
**SupraMarginal**	2	−60	−24	33	24	23	0.49	0.50	53	0.45	1.74	117	0.44
**Middle Temporal**	21	57	−39	−3	23	20	0.48	0.49	51	0.57	1.70	145	0.43
**Inferior Parietal**	3	−54	−24	45	24	20	0.43	0.49	53	0.50	1.71	124	0.42
**Inferior Frontal**	45	−51	15	27	24	19	0.47	0.49	55	0.57	1.73	150	0.48
**Anterior Cingulum**	10	−6	48	−3	23	18	0.50	0.48	51	0.57	1.63	110	0.34
**Anterior Cingulum**	10	−3	48	3	24	19	0.44	0.51	54	0.57	1.75	107	0.37
**Middle Frontal**	47	−36	51	0	23	16	0.48	0.51	49	0.58	1.61	89	0.43
**Middle Occipital**	19	−24	−87	21	23	18	0.50	0.52	54	0.50	1.66	127	0.36

ICC: two-way mixed single measures intraclass correlation, ICC(3,1).

T: Student's t-test.

### Reliability

Intraclass correlation analyses of test-retest datasets from children/adolescents demonstrated the good reliability of the *lC* and *lL* patterns across sessions and thresholds (Table 1). Specifically, the ICC(3,1) patterns in Figure 2B show that most regions with high *lC*, *lL* and *l*FCD also had, ICC(3,1) > 0.5. In particular, thalamus, visual cortex and posterior and anterior cingulum, striatum and midbrain exhibited ICC(3,1) > 0.6. The variability of the ICC coefficients with the 3×3 different thresholds used for the computation of the *lC* and *lL* maps was small (SD < 0.05). The average two-way mixed single (ICC(3,1)  =  0.46 for *lC*; 0.47 for *lL*; and 0.39 for *l*FCD) and average (ICC(3,k)  =  0.71 for *lC*; 0.70 for *lL*; and 0.62 for *l*FCD) measures in the whole brain were maximal for *R*_T1_ =  0.50 and *R*_T2_ =  0.65.

### Brain networks versus random networks: Small-worldness

**Figure 3:**
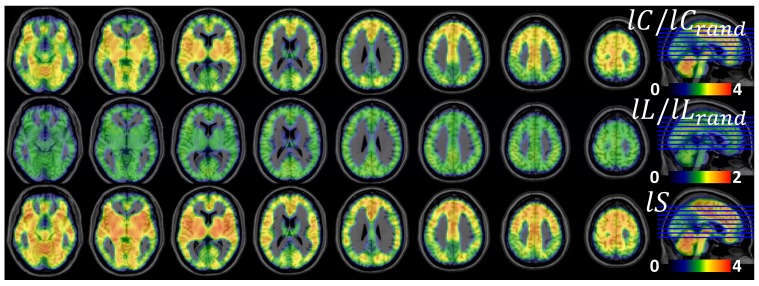
Regional measures of relative graph theory properties. Average measures of local clustering, *l*C/*l*C_rand_, and characteristic path length, *l*L/*l*L_rand_, relative to those of comparable random networks, and small-worldness, *lS*, for the networks functionally connected to each imaging voxel, superimposed on axial views of the human brain. Sample: 3 resting-state sessions from 40 healthy children (WashU dataset), correlation thresholds: *R*_T1_  =  0.40, 0.45 and 0.50; *R*_T2_  =  0.65, 0.70 and 0.75.

In children/adolescents, the local functional connectivity networks had higher clustering and similar characteristic path length, compared to random networks with the same number of nodes and edges. Precisely, 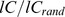, was maximal for subcortical regions (thalamus, caudate and putamen), cerebellum (hemispheres and vermis) and insula, regions that demonstrated > 3 times higher clustering than the comparable random networks (Fig 3 and Table 2). Motor and premotor cortical regions also showed higher clustering than the comparable random networks. However, the strength of the relative characteristic path length, 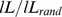, did not vary significantly across regions (Fig 3 and Table 2). Thus, the path length of the local functional connectivity networks was similar 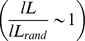 and did not demonstrate statistically significant *lL*-differences with comparable random networks in any brain region. Therefore, the distribution of the small-worldness peaked at the same regions as 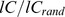 (Fig 3 and Table 2), revealing the small-world topology of the functional connectivity in subcortical and cerebellar regions. *lS* had significant variability across imaging voxels but low variability across subjects, as demonstrated by PCA (the principal component accounted for up to 87% of the variance of *lS*, Fig S2) and Student's t-tests (*t*-score > 20; Table 2). However, the reliability of the *lS* patterns across sessions and thresholds [ICC(3,1)  =  0.16 and ICC(3,k)  =  0.79; whole-brain average values] was markedly reduced compared to those of *lC*, *lL* and *l*FCD, probably reflecting the low reliability of the 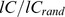 patterns [ICC(3,1)  =  0.17 and ICC(3,k)  =  0.80; whole-brain average values]. On the other hand, the reliability of the 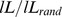 patterns [ICC(3,1)  =  0.57 and ICC(3,k)  =  0.97; whole-brain average values] was similar to that of *lC*, *lL* and *l*FCD. It is noteworthy that the main *l*FCD hub in the brain (ventral precuneus/posterior cingulum) had weaker *lS* than other cortical, subcortical or cerebellar areas (Fig 3), suggestive of a different pattern of organization in this brain region.

**Table 2:** Strength and statistical significance for local clustering (*lC*) and characteristic path length (*lL*), relative to comparable random networks, and for local small-worldness (*lS*) and degree for the MNI coordinates of *l*C/*l*C_rand_ local maxima from children/adolescents.

Region	BA/lobe	MNI coordinates [mm]			Clustering		Path Length		Small-worldness		Local degree	
		x	y	z	*lC/lC_rand_*	T	*lL/lL_rand_*	T	*lS*	T	lFCD (voxels)	T
**Cerebellum**	IV–V	−9	−48	−24	3.3	24	1.0	128	3.4	22	19	18
**Thalamus**		−12	−18	3	3.3	28	1.0	90	3.6	25	14	15
**Vermis**	IV–V	6	−54	−24	3.4	23	1.1	136	3.4	22	21	19
**Cerebellum**	VI	9	−63	−24	3.3	23	1.0	108	3.5	22	19	18
**Cerebellum**	VIII	−21	−51	−36	3.3	27	1.0	89	3.5	23	17	14
**Cerebellum**	VI	18	−54	−30	3.2	21	1.0	110	3.4	20	17	17
**Thalamus**		12	−15	3	3.2	25	1.0	92	3.5	22	14	16
**Paracentral**	4	−12	−33	63	3.1	24	1.0	122	3.3	21	18	18
**Insula**	47	36	15	0	3.2	29	1.0	102	3.5	26	12	26
**Paracentral**	6	−15	−12	66	3.1	25	1.0	117	3.3	23	17	16
**Insula**	13	−39	3	6	3.1	26	1.0	99	3.4	23	14	22
**Putamen**		27	−6	6	3.1	26	0.9	92	3.6	23	12	20
**Insula**	13	39	0	6	3.1	25	0.9	94	3.5	23	12	24
**Caudate/Putamen**		15	12	3	3.0	26	1.0	93	3.2	23	15	19

*l*FCD: Functional connectivity density. T: Student's t-test. Sample: WashU (40 healthy subjects, 3 ‘resting-state’ MRI sessions). ROI analysis: Average values in 9-mm cubic ROIs (27 voxels) centered at the MNI coordinates of the local maxima.

### ROI analyses

**Figure 4:**
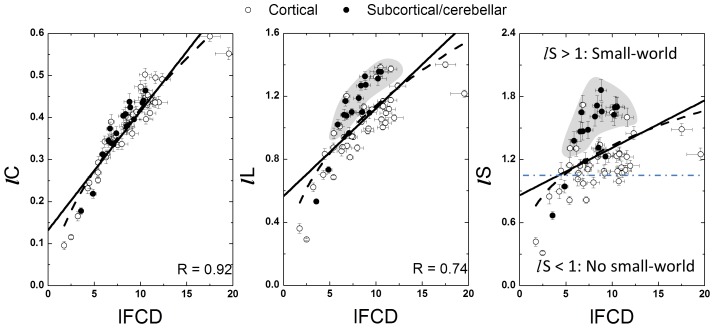
Association between graph-theory measures from anatomical ROIs. Scatter plots showing the linear (solid line) and non-linear (negative power scaling, dashed line) associations of *l*FCD with *lC*, *lL* and *lS*. Shaded highlights identify regions with higher small-worldness than average for a given local degree. The dash dot line indicates the boundary of the small-world topology (*lS*  =  1). Sample: 40 healthy children (WashU dataset), correlation thresholds: *R*_T1_  =  0. 50; *R*_T2_  =  0.65.

**Figure 5:**
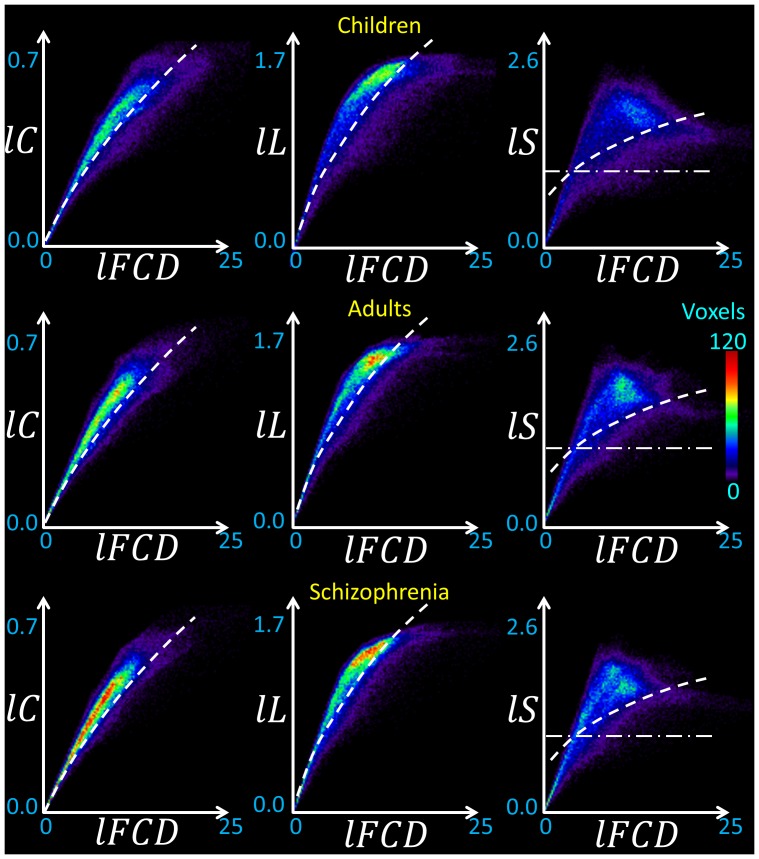
Voxelwise association between graph-theory measures. Two-dimensional histograms (probability distributions) corresponding to (non-linear) relationships between voxelwise measures of *l*FCD, *lC*, *lL* and *lS*, averaged across children (N  =  40), adults (N  =  74) and schizophrenia patients (N  =  69). The color maps (frequency) reflect the number of voxels within each of the 128 × 128 uniform 2d-intervals (bins). Dashed lines reflect the power scaling in Fig 3. The dash dot line indicates the boundary of the small-world topology (*lS*  =  1). Correlation thresholds: *R*_T1_  =  0. 50; *R*_T2_  =  0.65.

Since Pearson correlations across the hub regions (Table 1) demonstrated a strong linear association between *lC* and *l*FCD [*lC*  =  (0.0056 ± 0.0008) × *l*FCD + (0.36 ± 0.02); R  =  0.91] and between *lL* and *l*FCD [*lL*  =  (0.0054 ± 0.0024) × *l*FCD + (1.56 ± 0.07); R < 0.55], we quantified *lC*, *lL*, *lS* and *l*FCD in anatomically defined ROIs in cortical, subcortical and cerebellar regions (Table S1). Linear regressions demonstrated the strong association of *l*FCD with *lC* and *lL* [*lC*  =  (0.028 ± 0.002) × *l*FCD + (0.13 ± 0.02); R  =  0.91] and between *lL* and *l*FCD [*lL*  =  (0.056 ± 0.007) × *l*FCD + (0.56 ± 0.06); R  =  0.74] such that brain regions with stronger *l*FCD also exhibited higher *lC* and longer *lL* (Fig 4). Note that non-linear power scaling curves also fitted the *lC* vs. *l*FCD and the *lL* vs. *l*FCD datasets (R^2^ > 0.66; Fig 4, dashed lines). However, *l*FCD and *lS* showed much weaker linear [*lS*  =  (0.045 ± 0.01) × *l*FCD + (0.86 ± 0.11); R  =  0.44] and non-linear (R^2^  = 0.29) associations; *lS* was maximal for globus pallidus, which exhibited moderate *l*FCD and was not considered a hub region in this study; other brain regions with high *lS* (> 1.4) were cerebellum (lobes III, IV-V, VI, VIII, IX and vermis), anterior and posterior cingulum (BA 23, 24 and 32), hippocampus, amygdala and thalamus, posterior transverse temporal (BA 42), retrosplenial and entorhinal cortices (BAs 30 and 34). Seven cortical areas (BAs 24, 27, 32, 34, 38 and 42), 6 subcortical areas (thalamus, putamen, globus pallidus, hippocampus, parahippocampus, amygdala) and the cerebellar lobes (III, IV-V, VI, VIII, IX) and cerebellar vermis showed higher *lS* for a given *l*FCD than the corresponding linear fit (Fig 4 shaded highlight), suggesting special (perhaps small-world) network topology in these regions. The same regions also showed longer *lL* for a given *l*FCD than the corresponding linear fit. The 2-dimensional density functions (histograms) in Fig 5 demonstrate similar relationships between *l*FCD, *lC*, *lL* and *lS* at the voxel level.

### Adults

The spatial distributions of the *lC*, *lL, lS* and *l*FCD measures in the healthy adults and schizophrenia patients were similar to those in children/adolescents (Figs 5 and S3). Specifically, in healthy adults and schizophrenia patients, *lC* and *l*FCD were high in posterior cingulum/ventral percuneus, cerebellum, and visual and parietal cortices; *lL* was high in cerebellum, posterior and anterior cingulum, and inferior frontal and inferior parietal cortices; and *lS* was high in putamen, insula, inferior and superior frontal cortex, anterior cingulum cerebellum, thalamus and hippocampus (Tables 3, S2 and S3).

**Table 3:** Statistical significance for differences in graph theory measures between children and adults.

Region	BA	MNI coordinates [mm]			Children > Adults [T]			
		x	y	Z	*lC*	lL*	*lS*	lFCD
Cuneus	19	3	−90	36	8.1	9.5	4.8	7.0
Postcentral	3	30	−39	66	2.6	7.9	NS	3.3
Thalamus		3	−9	9	NS	6.3	4.7	NS
Superior Frontal	11	−21	66	0	4.8	6.2	4.3	7.3
Lingual	19	−36	−93	−21	NS	6.0	5.6	5.6
Superior OFC	11	15	66	−3	4.3	5.9	3.1	5.5
Temporal Pole	38	45	18	−30	3.8	5.3	NS	4.0
Inferior Temporal	37	48	−63	−12	4.2	4.5	NS	3.0
Middle Frontal	46	36	48	24	5.1	5.7	NS	4.7
Inferior Frontal	45	48	27	21	5.6	6.3	NS	5.8
Inferior Frontal	45	45	33	15	4.0	5.5	NS	4.6
Rectus	11	6	36	−15	5.9	5.7	NS	3.9
Rectus	11	−6	39	−18	5.3	4.3	NS	5.0
Middle Frontal	46	−24	54	30	5.3	4.4	NS	5.4

Sample: 40 healthy children and 74 healthy adults. *l*C: local clustering; *l*L: local characteristic path length; *l*S: local small-worldness; *l*FCD: local degree. *One way ANOVA: P_FWE_ < 0.05 cluster-level corrected. SPM8 model: one-way ANOVA with gender and motion covariates.

### Brain maturation

**Figure 6:**
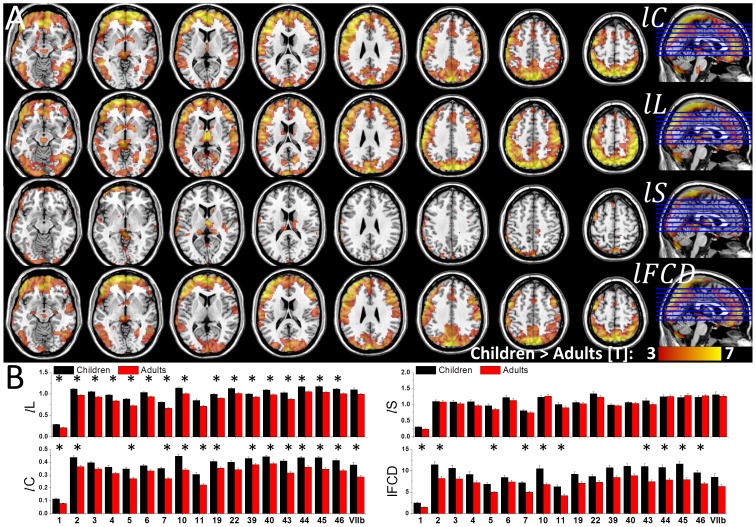
Pruning of functional connectivity during brain maturation. (A) Statistical significance of higher graph theory measures for 40 children than for 74 adults superimposed on axial views of the human brain. (B) Average values of the graph theory measures in anatomical ROI that demonstrated significant differences in the measures between children and adults. X-axis labels: Brodmann Areas (number labels), thalamus (THA), putamen (PUT), globus pallidus (GP), amygdala (AMY) and the posterior lobe of the cerebellum (lobe X). Correlation thresholds: *R*_T1_  =  0. 50; *R*_T2_  =  0.65.

Direct comparison of graph theory metrics between children/adolescents and adults showed that children/adolescents had longer *lL*, higher *lC* and stronger *l*FCD in cortical regions and longer *lL* and higher *l*C and *lS* in thalamus than adults (P_FWE_ < 0.05; Fig 6A and Table 3). However, group differences in *l*FCD for the thalamus did not reach statistical significance. Similarly group differences in *lS* for cuneus, postcentral, inferior, middle and superior frontal gyri did not reach statistical significance. The anatomical ROI analysis confirmed the higher *lC*, longer *lL* and stronger *l*FCD in language areas (BAs 44, 45 and 46) for children/adolescents than for adults (P_c_ < 0.05, Bonferroni corrected for multiple comparisons; Fig 6B). In addition, the average values in somatosensoty (BAs 1, 2 and 5) and gustatory (BA 43) areas, superior parietal (BA 7), frontopolar and rostrolateral prefrontal (BA 10) cortices (*lC*, *lL* and *l*FCD), lateral parietal (BAs 39 and 40) and association visual (BA 19) cortices (*lC* and *lL*), rectus (BA 11) (*lC* and *l*FCD), motor (BA 4) and premotor (BA 6) and auditory (BA 22, Heschl's gyrus) areas (*lL*) and cerebellum (VIIb lobe; *lC*) were higher for children/adolescents than for adults (P_c_ < 0.05; Fig 6B). No anatomical region showed significant differences in small-worldness between children/adolescents and adults.

### Schizophrenia

**Figure 7:**
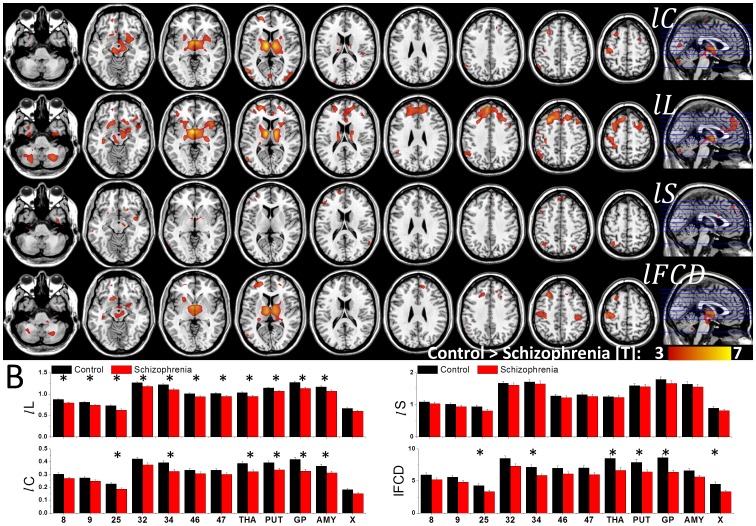
Reduced clustering, path length and local degree in schizophrenia. (A) Statistical significance of lower graph theory measures for 69 schizophrenia patients than for 74 healthy controls superimposed on axial views of the human brain. (B) Average values of the graph theory measures in anatomical ROI that demonstrated significant differences in the measures between patients and controls. X-axis labels: Brodmann Areas (number labels), thalamus (THA), putamen (PUT), globus pallidus (GP), amygdala (AMY) and the posterior lobe of the cerebellum (lobe X). Correlation thresholds: *R*_T1_  =  0. 50; *R*_T2_  =  0.65.

Patients with schizophrenia had shorter *lL* in thalamus, putamen, anterior insula, midbrain, middle frontal, postcentral and angular gyri and cerebellum (VIIb, VIII), and lower *lC* and weaker *l*FCD in thalamus, putamen, anterior insula, midbrain and postcentral gyrus than healthy controls (P_FWE_ < 0.05; Fig 7A and Table 3). On the other hand, differences in small-worldness between patients and controls were not statistically significant for any brain region. No brain region showed significant differences in small-worldness between patients and controls.

### Thalamic and midbrain hypo connectivity in schizophrenia patients

**Figure 8:**
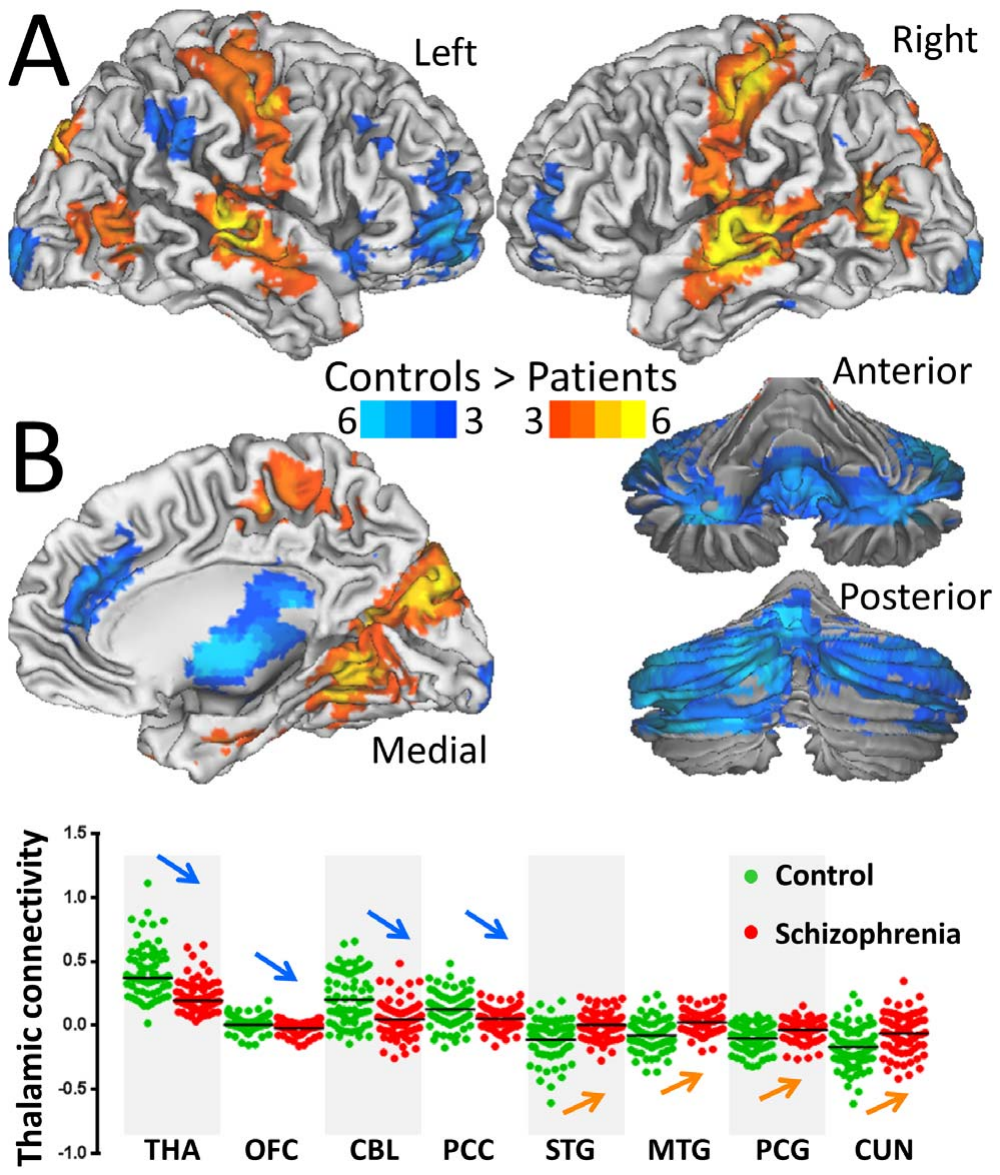
Reduced functional connectivity of the thalamus in schizophrenia. (A) Statistical significance (t-scores) of decreases in the positive (blue-cyan) and negative (red-yellow) FC of the thalamus in schizophrenia (N  =  69) compared to healthy controls (N  =  74), superimposed on lateral and medial surface views of the cerebrum and anterior and posterior surface views of the cerebellum. (B) Average ROI measures of thalamic FC across subjects for selected ROIs (0.9 mm cubes; 27 voxels) centered at the MNI coordinates listed in Table S3, showing the lower absolute FC for schizophrenia patients than for controls in thalamus (THA), orbitofrontal cortex (OFC; BA 11), posterior lobe of the cerebellum (CBL), posterior cingulum (PCC) (positive connectivity: blue arrows; P < 0.0005), and for superior and middle temporal (STG and MTG; BA 22) and postcentral (PCG; BA 3) gyri and cuneus (BA 19) (negative connectivity: orange arrows; P < 0.0005). MNI coordinates of the bilateral cubic seed (54 voxels) in the thalamus: *x*  =  ±6 mm; *y*  =  −3 mm; and *z*  =  0 mm.

**Figure 9:**
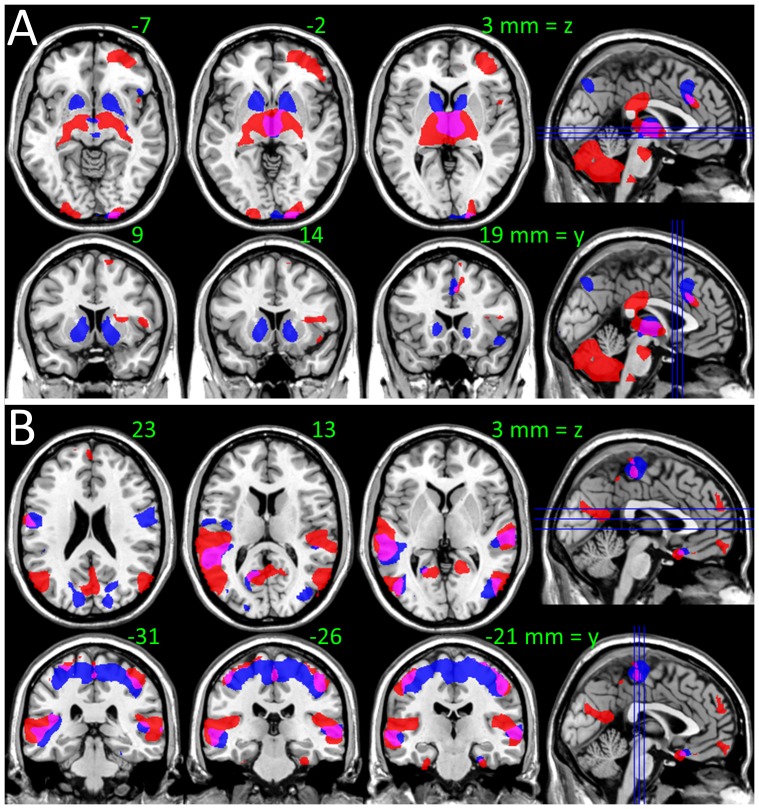
Overlapping hypo connectivity patterns for thalamus and midbrain in schizophrenia. Overlap and distinct patterns of hypo connectivity with thalamus (red) and SN (blue) for 69 schizophrenia patients versus 74 healthy controls at P < 0.001, superimposed on axial and coronal views of the human brain. The weaker positive (A) and negative (B) connectivity patterns overlap (pink) in thalamus, BAs 2–4, 6, 17–19, 21, 23, 24, 36, 37, 43 and differentiate in ventral striatum, putamen, pallidum (blue), cerebellum, BAs 10, 11, 32, 40 and 44–47 (red).

We mapped the networks functionally connected to the bilateral thalamus and midbrain clusters (SN) in Table 4 to map the hypo connectivity pathways in schizophrenic patients. Specifically, seed-voxel correlations revealed the patients had lower positive thalamic FC in cerebellum, thalamus, pons, cingulum, and orbitofrontal, inferior frontal and occipital cortices, and lower negative thalamic FC in auditory (Heschl's gyrus), visual, motor, premotor and somatosensory cortices (P_FWE_ < 0.05; Fig 8 and Table S3). Seed-voxel correlations also revealed that patients had lower positive SN connectivity with thalamus, putamen and cerebellum, and lower negative SN connectivity with premotor, auditory and visual areas (Fig S4). The patient's hypo connectivity patterns for thalamus and SN overlapped in thalamus and anterior cingulum (Fig 9A, pink), motor, somatosensory, auditory and visual areas (Fig 9B, pink). In ventral striatum, putamen and posterior parietal cortex (Fig 9A, blue), motor and premotor and visual cortices (Fig 9B, blue) the patient's hypo connectivity was stronger for SN than for thalamus (P_FWE_ < 0.05). In cerebellum, posterior cingulum, pons, lateral orbitofrontal and inferior frontal cortices (Fig 9A, red), middle cingulum, posterior insula and the occipitotemporal area 37 (Fig 9B, red) the patient's hypo connectivity was stronger for thalamus than for SN (P_FWE_ < 0.05).

**Table 4:** Statistical significance for differences in graph theory measures between schizophrenia patients and controls.

Region	BA/lobe	MNI coordinates [mm]			Control > Schizophrenia [T]			
		x	y	z	*lC*	lL*	*lS*	lFCD (voxels)
**Thalamus**		−6	−3	0	6.1	8.1	2.9	5.6
**Thalamus**		15	−9	12	5.7	5.6	1.9	4.4
**Thalamus**		6	−3	0	6.3	6.1	3.0	5.5
**Midbrain**		12	−15	−12	3.9	2.9	2.1	3.2
**Middle Frontal**	8	−24	27	48	2.8	5.0	NS	4.2
**Middle Frontal**	10	−24	54	12	4.1	4.3	NS	3.9
**Superior Middle Frontal**	9	−6	48	36	1.8	4.4	NS	2.2
**Cerebellum**	VIII	27	−63	−42	3.5	4.1	NS	3.7
**Angular**	39	−48	−54	33	NS	3.7	NS	NS
**Cerebellum**	VIII	−39	−57	−48	2.6	3.0	NS	2.8
**Cerebellum**	VIIb	−24	−69	−42	2.7	3.0	NS	2.5

Sample: 69 patients and 74 matching controls. *l*C: local clustering; *l*L: local characteristic path length; *l*S: local small-worldness; *l*FCD: local degree. *One way ANOVA: P_FWE_ < 0.05 cluster-level corrected. SPM8 model: one-way ANOVA with gender, age and motion covariates.

## Discussion

Global network properties are insensitive to regional abnormalities. Here we propose a novel ultra-fast (15 minutes/subject) methodology for mapping regional properties of network topology (*lC*, *lL*, *lS*, and *l*FCD) at 3-mm isotropic resolution, a methodology that allowed us to assess 57,713 different brain networks (12 ± 6 nodes per network) for each of the 183 subjects in the study. The proposed voxelwise methodology is based on 2 separate correlations thresholds, 0.5 was used to compute *l*FCD and to define the local network connected to each individual voxel, and 0.65 was used to compute regional graph theory metrics (*lC*, *lL* and *lS*). Note that this approach restricted *lC, lL* and *lS* to the local network functionally connected to each voxel, not to the whole brain. The method revealed that strongly connected hub regions in posterior cingulum, parietal, prefrontal and occipital cortices also have higher *lL* and *lC* than weakly connected brain regions (Fig 2). This finding is consistent with the linear scaling of small-worldness with network size [17], [34]. Our results are also consistent with previous functional connectivity studies based on 90 anatomical regions [6] or 75 independent components [19], which reported whole-brain 0.2 ≤ *C* < 0.23 and 0.2 ≤ *L* < 0.23, and with studies on the anatomical connectivity in the macaque visual cortex [35], [36] but less so with results derived from magnetoencephalographic (MEG) data in the 2–37.5 Hz bandwidth [4]. Specifically, Basset et al. documented for the whole-brain 0.2 ≤ *C* < 0.23, which did not differ from the whole-brain average clustering coefficient in this study (<*lC*>  =  0.35 ± 0.12), and 4.5 ≤ *L* < 5.2 which is significantly higher than the whole-brain average characteristic path length in this study (<*lL*>  =  1.25 ± 0.40). These differences could reflect methodological differences between MEG (electrophysiological signals) and MRI (hemodynamic signals) as well as differential network analysis (global versus local networks). We show also that the graph theory properties of the brain networks were not constant but vary significantly across regions, partially reflecting the spatial variability of *l*FCD. Specifically, across the anatomical ROIs the variability in these properties reached tenfold (0.1 < *lC* < 0.59; 0.3 < *lL* < 1.4; 0.3 < *lS* < 1.9; and 2 < *l*FCD < 20).

### Reliability

Intraclass correlation demonstrated the good reliability of the graph theory measures across subjects, sessions and parameters of the method (thresholds), particularly for basal ganglia, thalamus, visual cortex and posterior and anterior cingulum (ICC(3,1) > 0.6). Overall, taking into account that the reliability of blood-oxygenation-level-dependent (BOLD) functional MRI studies typically ranges between 20% and 80% [37]–[39], the test-retest reliability of *lL*, *lC* and *l*FCD measures is similar to that of standard functional imaging techniques. The low average reliability of the *lS* (two-way mixed single measures ICC  =  0.16), which probably reflects the variability induced by the clustering of the random network, suggests high within-subjects variability and low statistical power for *lS*, which could explain the lack of significant effects of brain maturation and schizophrenia for *lS* in this study. Furthermore, the principal component accounted for a large fraction of the variance, demonstrating the low between-subjects variability of the proposed voxelwise graph theory measures. Across subjects, the variability of *lL* (6%) was lower than that of *lC* (13%), *lS* (29%) and *l*FCD (31%), suggesting lower influence of demographics (age, gender), state (alertness and fatigue) and/or genetics factors for *lL* than for the other graph theory measures.

### Small-worldness

Traditionally, the small-worldness coefficient *S* is considered a measure of the balance of segregation and integration in a functional/structural network. However, *S* is a global property of the brain, which is not sensitive to regional abnormalities. Here we define a local measure of small-worldness (*lS*) in analogy with the global measure of *S* [17]. Thus *lS* reflects the efficiency of regional segregation and integration for neural communication in the local networks. The small-world topology (*C*/*C*_rand_ > 1; *L*/*L*_rand_ ∼1), as revealed by *lS*, was more frequent in subcortical regions (globus pallidus, cerebellum, thalamus, hippocampus, amygdala and midbrain), cingulum (BAs 30, 23, 24 and 32) and temporal (BAs 34 and 42) cortices than in other cortical regions (Figs 3 and 4). We hypothesized that the functional connectivity of the posterior ventral parietal and occipital regions would demonstrate a small-world topology characterized by higher clustering and similar path length, compared to random networks because these hubs are heavily interconnected [40] and energy demanding [41]. Note that small-world networks achieve high communication efficiency through few random connectivity changes between nodes that retain the high clustering of the regular networks and the short characteristic path length of the random networks [1]. Indeed, *lL*/*lL*_rand_ ∼ 1 for the hubs in ventral and posterior parietal and occipital cortices, regions that had high *lC*. However, whereas the main functional connectivity hub of the brain (BA 23) had high *lC*/*lC*_rand_, which demonstrated its small-world properties, most cortical hub regions did not show high *lC*/*lC*_rand_, which is not consistent with their small-world topology. Moreover the ventral precuneus/posterior cingulum showed weaker *lS* than other cortical, subcortical or cerebellar regions. The significance of this unique pattern is unclear but is consistent with prior findings showing that the precuneus is a brain region with much greater heterogeneity than other cortical regions [42]. Overall these findings highlight the variability of *lS* in the human brain but its potential impact on functional connectivity could be limited by its low reliability.

### Maturation

Local clustering, path length and degree were higher for children than for adults in most cortical regions (including language areas, orbitofrontal, and inferior temporal and occipital cortices) and thalamus, suggesting that functional connectivity decreases in the transition into adulthood. Indeed histological studies from postmortem brains show that synaptic density in the cortex is maximal at 2–4 years of age (double adult levels), with significant pruning occurring in the transition from adolescence into adulthood [43]. These findings are also consistent with the higher functional connectivity [21], [44]–[46] and the lower levels of hierarchical brain network organization for children than for adults [47]. It has been proposed that connectivity decreases from childhood to adulthood reflect less specialized or efficient functional networks in children as brain networks develop from a local to a distributed organization with decreased short-range connections [48], [49]. Indeed, the proposed graph theory measures (*lC*, *lL* and *l*FCD) reflect the local connectivity of the brain regions and their decreases from childhood to adulthood reveal decreased short-range connectivity and weaker local small-world topology. These findings support the thoughts that rapid changes in human brain circuitry occur from childhood to adulthood, which could explain the increased vulnerability to risk-taking behaviors [50] and to psychiatric disorders such as schizophrenia [51], [52] during this transitional stage.

### Schizophrenia

The local graph theory metrics (*lC*, *lL* and *l*FCD) were weaker for patients with schizophrenia than for healthy controls, consistently with the disconnection hypothesis of schizophrenia [53], which postulates that hallucinations, delusions, loss of initiative, and cognitive dysfunction result from abnormal wiring of brain networks [54]. Indeed, previous imaging studies have reported lower regional brain connectivity [16], loss of integrity of frontal and temporal white matter connections [55]–[57] and altered default mode network connectivity [58]–[61] in patients with schizophrenia. More recently, reduced interconnectivity among hubs has been reported in patients with schizophrenia [62]. The present study reveals lower degree of connectivity, clustering and path length in schizophrenia that was most prominent in the thalamus and the midbrain. This is consistent with our prior findings of thalamic and midbrain disconnection in patients with schizophrenia [16], [63]. Compared to controls the patients demonstrated lower thalamic connectivity in cerebellum, cingulum, frontal, visual, auditory, motor and premotor cortices, which is consistent with the reduced connectivity between thalamus and prefrontal cortex in schizophrenia patients [64], [65]. In addition, the present study demonstrates lower midbrain (centered in SN) connectivity in thalamus and putamen for patients than for controls, which is consistent with our previous studies on the functional connectivity of SN [21] and with the hypoconnectivity of midbrain with thalamus and putamen in unmedicated patients with schizophrenia [63]. The prefrontal cortex also showed significantly shorter *lL* in schizophrenic patients than controls, which is also consistent with reports of reduced connectivity of the prefrontal cortex in schizophrenia [66], [67]. Interestingly, decreased metabolic activity in frontal cortex as well as decreased frontal blood flow was one of the first brain imaging findings reported in schizophrenia [16], [68], [69], which might reflect in part reduced prefrontal connectivity since regional connectivity density is linearly correlated with metabolic rate [41]. Similarly the findings of reduced connectivity in left superior temporal cortex (Wernicke's area) in schizophrenia corroborate prior findings of decreased connectivity of the planum temporale (superior temporal cortex within the Sylvian fissure, posterior to the auditory cortex) in schizophrenia, which was associated with psychopathology [70]. Since the superior temporal cortex is involved in the generation of hallucinations [71] the reduced connectivity with thalamus could underlie hallucinatory behaviors. The findings in schizophrenic patients contrast with the increases in *lC*, *lL* and *l*FCD in children when compared to adults and suggest that they might reflect enhanced pruning in schizophrenic patients. Indeed synaptic pruning has been hypothesized to underlie the neuropathology of schizophrenia [72].

### Study limitations

Information about the use of antipsychotic medications was not available in this work, which is a confounding factor for the interpretation of the hypoconnectivity we observed in schizophrenic patients. Previous studies have shown that resting state fronto-temporal functional connectivity was increased in schizophrenia patients compared to controls at baseline and subsequently normalized in patients after six weeks of antipsychotic treatment [73]. Furthermore, an acute dose of sulpiride, a selective dopamine D2 receptor antagonist was shown to affect global and local efficiency of brain networks [74], and the disruption of small-world properties was correlated with the chlorpromazine equivalent dose that the patients were receiving in one resting-state study [75]. Thus, further studies are needed to understand the contribution of neuroleptic medications on the decreased connectivity that we and others are reporting in the brain of schizophrenic patients.

### Summary

Here we propose novel ultra-fast graph theory measures to map local network properties such as *lC*, *lL* and *lS* at 3-mm isotropic resolution in the human brain. These measures exhibited good reliability across test-retest measures and different computational parameters for most brain regions (*lC* and *lL*: ICC(3,1) > 0.5) and low variability across subjects (< 29%). Subcortical regions (globus pallidus, thalamus, hippocampus and amygdala), cerebellum, cingulum and temporal cortex demonstrated stronger small-world topology than other brain regions. The present study reveals higher *l*FCD, *lC* and *lL* values in cortical regions for children/adolescents than adults suggesting maturation effects on the small-world topology of the local connectivity and lower *l*FCD, *lC* and *lL* values in thalamus and midbrain for patients with schizophrenia than for controls, suggesting exaggerated pruning of connectivity in these brain regions.

## Supporting Information

Figure S1 **Principal component analysis for local measures of clustering,** ***lC*** **, and characteristic path length,** ***lL*** **, showing that the principal component (PC0) captured a large fraction of the variance (> 87%).** Sample: 40 healthy children (WashU dataset), correlation thresholds: *R*_T1_  =  0. 50; *R*_T2_  =  0.65.
(TIF)

Figure S2 **Principal component analysis for local small-worldness,** ***lS*** **, and local functional connectivity density,** ***l*** **FCD, showing that the principal component (PC0) captured a large fraction of the variance (> 69%).** Sample: 40 healthy children (WashU dataset), correlation thresholds: *R*_T1_  =  0. 50; *R*_T2_  =  0.65.
(TIF)

Figure S3 **Average strength of local measures of clustering,** ***lC*** **, characteristic path length,** ***lL*** **, small-worldness,** ***lS*** **, and functional connectivity density (degree),** ***l*** **FCD, of the networks functionally connected to each imaging voxel for 74 healthy adults (A) and 69 schizophrenia patients (B), superimposed on axial views of the human brain.**
(TIF)

Figure S4 **Statistical significance (t-scores) of decreases in the positive (blue-cyan) and negative (red-yellow) FC of the SN seed between patients with schizophrenia (N  =  69) and healthy controls (N  =  74), superimposed on lateral and medial surface views of the cerebrum and anterior and posterior surface views of the cerebellum.**
(TIF)

Table S1 **Strength of the local degree (lFCD), clustering (lC), characteristic path length (lL) and small-worldness (lS) at the locations of the lFCD-hubs in the brain of schizophrenia patients (SCZ) and controls (CON).**
(DOCX)

Table S2 **Average values in anatomical ROIs for local clustering (lC), characteristic path length (lL), small-worldness (lS) and local degree (lFCD) from children/adolescents.**
(DOCX)

Table S3 **Average values in anatomical ROIs for local clustering (lC), characteristic path length (lL), small-worldness (lS) and local degree (lFCD) for healthy adult (CON) and schizophrenia patients (SCZ).**
(DOCX)
