# Supplementary figures and images for: Mapping Small-World Properties through Development in the Human Brain: Disruption in Schizophrenia
Source: PLoS One. 2014 Apr 30;9(4):e96176. doi: 10.1371/journal.pone.0096176 (PMC4005771; doi:10.1371/journal.pone.0096176)

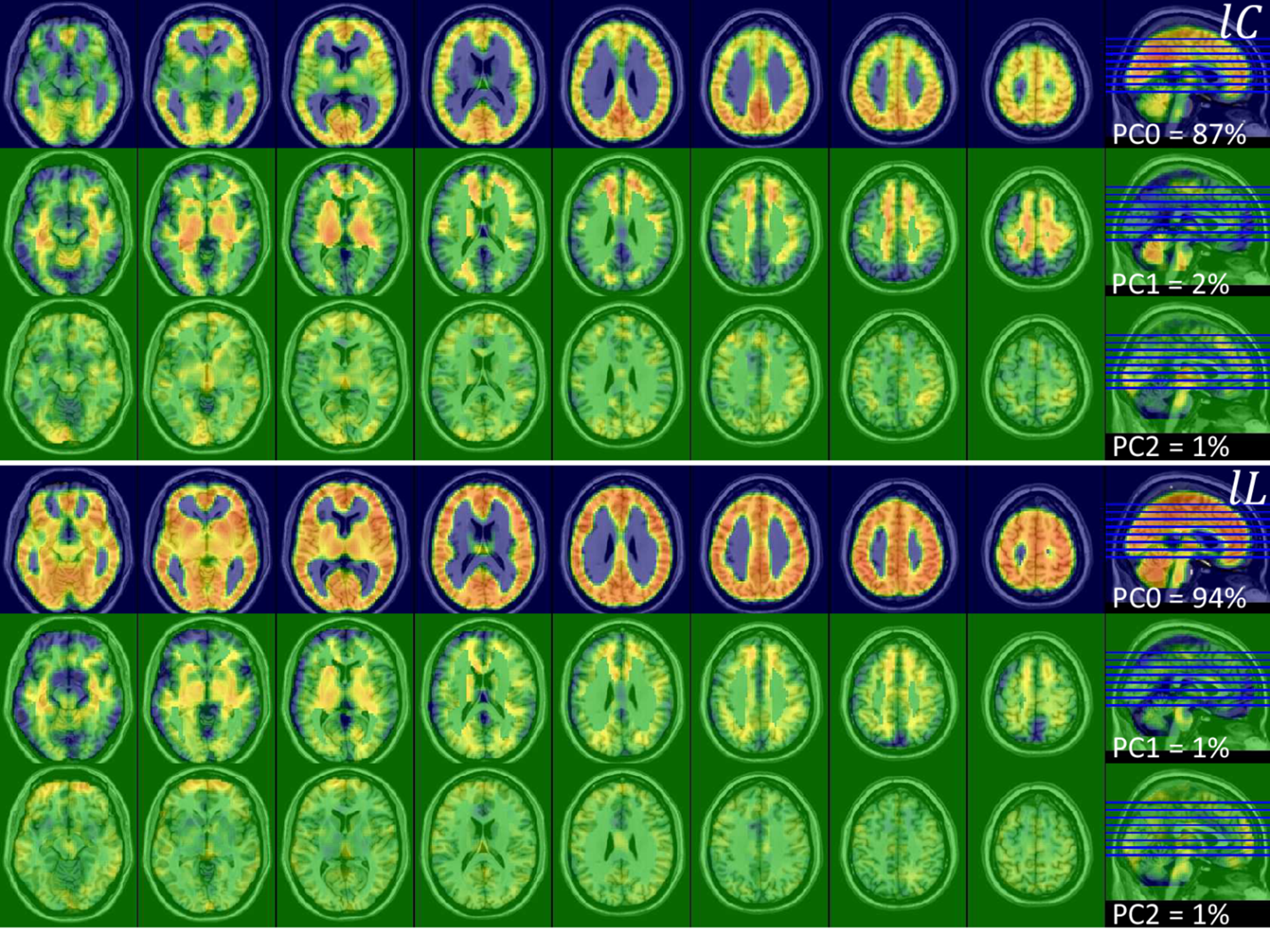

Supplement: Figure S1 — Principal component analysis for local measures of clustering, lC , and characteristic path length, lL , showing that the principal component (PC0) captured a large fraction of the variance (> 87%). Sample: 40 healthy children (WashU dataset), correlation thresholds: R T1 = 0. 50; R T2 = 0.65. (TIF) [file pone.0096176.s001.tif]

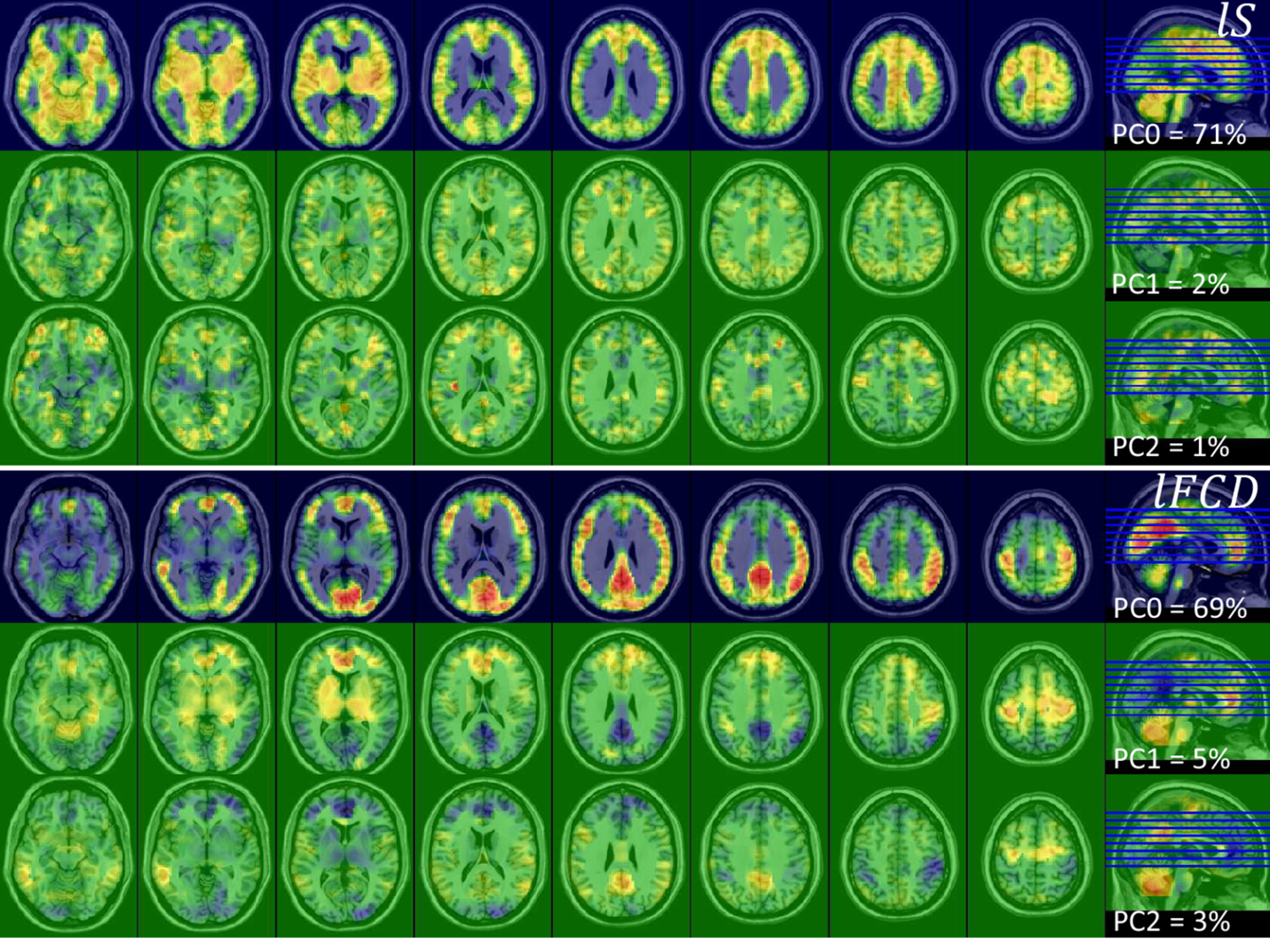

Supplement: Figure S2 — Principal component analysis for local small-worldness, lS , and local functional connectivity density, l FCD, showing that the principal component (PC0) captured a large fraction of the variance (> 69%). Sample: 40 healthy children (WashU dataset), correlation thresholds: R T1 = 0. 50; R T2 = 0.65. (TIF) [file pone.0096176.s002.tif]

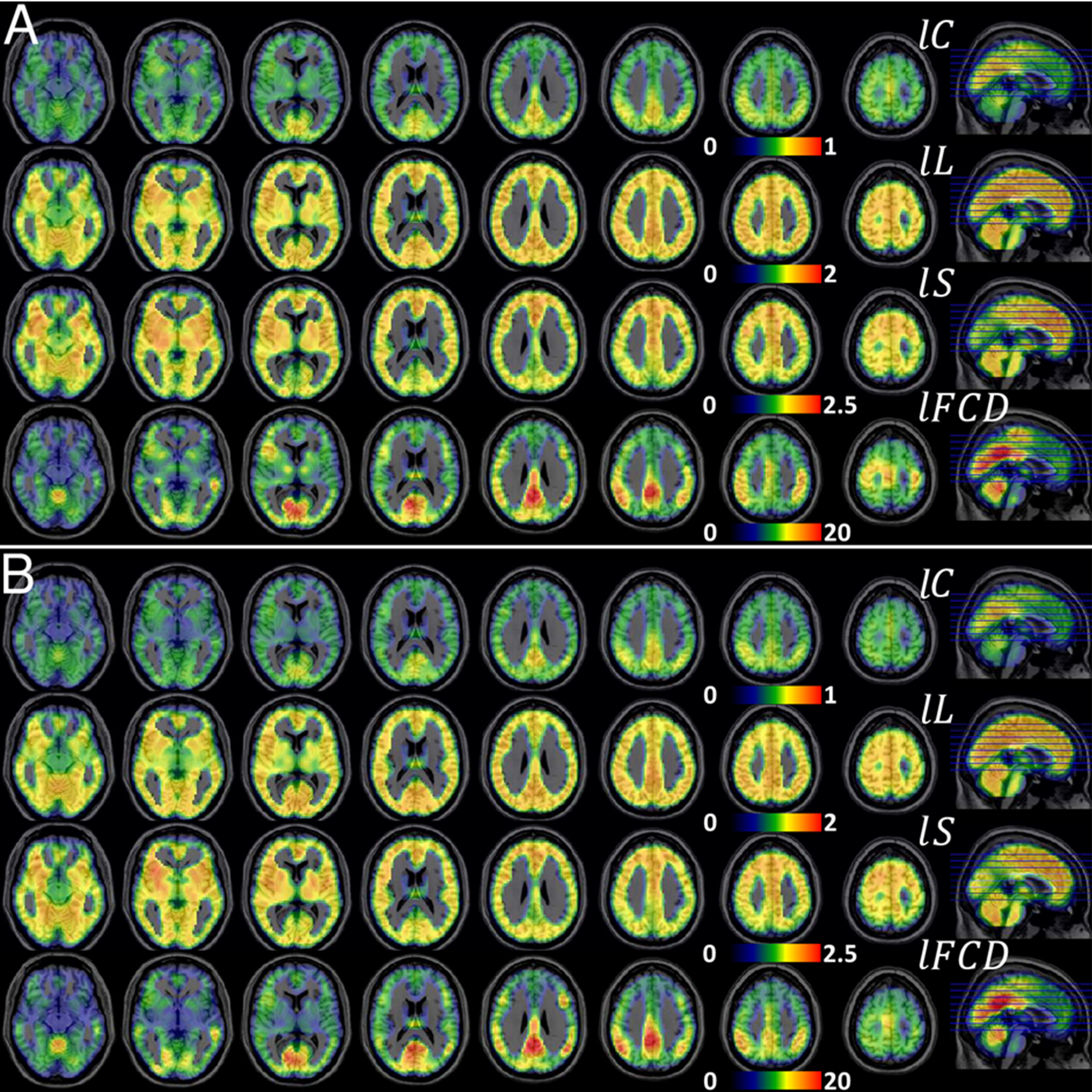

Supplement: Figure S3 — Average strength of local measures of clustering, lC , characteristic path length, lL , small-worldness, lS , and functional connectivity density (degree), l FCD, of the networks functionally connected to each imaging voxel for 74 healthy adults (A) and 69 schizophrenia patients (B), superimposed on axial views of the human brain. (TIF) [file pone.0096176.s003.tif]

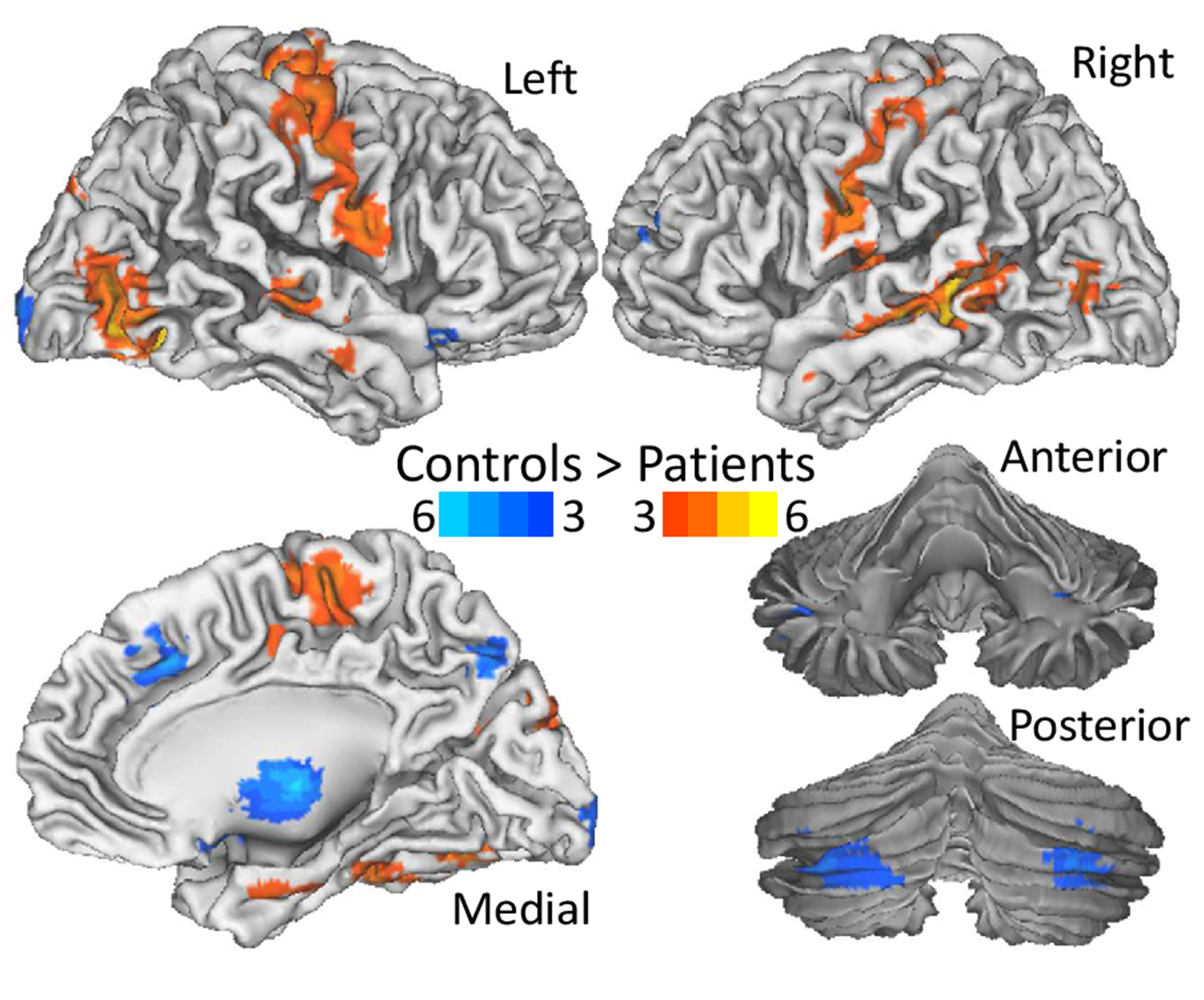

Supplement: Figure S4 — Statistical significance (t-scores) of decreases in the positive (blue-cyan) and negative (red-yellow) FC of the SN seed between patients with schizophrenia (N = 69) and healthy controls (N = 74), superimposed on lateral and medial surface views of the cerebrum and anterior and posterior surface views of the cerebellum. (TIF) [file pone.0096176.s004.tif]
