# Supplementary material for: Mapping Small-World Properties through Development in the Human Brain: Disruption in Schizophrenia
Source: PLoS One. 2014 Apr 30;9(4):e96176. doi: 10.1371/journal.pone.0096176 (PMC4005771; doi:10.1371/journal.pone.0096176)
Supplement: Table S1 — Strength of the local degree (lFCD), clustering (lC), characteristic path length (lL) and small-worldness (lS) at the locations of the lFCD-hubs in the brain of schizophrenia patients (SCZ) and controls (CON). (DOCX) [file pone.0096176.s005.docx]

| # | Region | BA  Nucl | MNI co [mm] | | | lFCD | | *l*C | | *l*L | | *l*S | |
| --- | --- | --- | --- | --- | --- | --- | --- | --- | --- | --- | --- | --- | --- |
|  |  |  | x | y | z | CON | SCZ | CON | SCZ | CON | SCZ | CON | SCZ |
| 1 | Post Cingulum | 23 | -6 | -48 | 33 | 27.3±1.7 | 25.9±1.5 | 0.65±0.01 | 0.64±0.01 | 1.51±0.01 | 1.49±0.01 | 1.31±0.03 | 1.34±0.03 |
| 2 | Calcarine | 17 | -12 | -69 | 12 | 20.3±1.3 | 18.2±1.4 | 0.59±0.01 | 0.55±0.01 | 1.38±0.02 | 1.45±0.02 | 1.40±0.03 | 1.49±0.05 |
| 3 | Vermis | IV-V | 3 | -51 | -21 | 18.9±1.2 | 16.8±1.1 | 0.58±0.02 | 0.55±0.02 | 1.52±0.02 | 1.51±0.02 | 1.68±0.06 | 1.69±0.07 |
| 4 | Cerebellum | IV-V | -6 | -51 | -21 | 18.6±1.2 | 16.9±1.1 | 0.57±0.02 | 0.55±0.02 | 1.52±0.02 | 1.51±0.02 | 1.71±0.06 | 1.73±0.08 |
| 5 | Angular | 39 | -51 | -57 | 39 | 17.6±1.0 | 16.6±1.0 | 0.58±0.01 | 0.57±0.01 | 1.40±0.01 | 1.35±0.02 | 1.29±0.03 | 1.23±0.02 |
| 6 | Angular | 39 | 48 | -60 | 30 | 16.9±0.9 | 16.0±1.0 | 0.60±0.01 | 0.58±0.01 | 1.42±0.01** | 1.36±0.01 | 1.30±0.02 | 1.32±0.03 |
| 7 | Middle Cingulum | 23 | 0 | -24 | 27 | 17.2±1.2 | 14.9±1.0 | 0.58±0.01 | 0.55±0.01 | 1.38±0.02 | 1.35±0.02 | 1.42±0.03 | 1.42±0.04 |
| 8 | Inferior Parietal | 40 | 39 | -48 | 45 | 14.6±0.7 | 13.5±0.6 | 0.55±0.01 | 0.54±0.01 | 1.43±0.01 | 1.38±0.02 | 1.42±0.04 | 1.40±0.03 |
| 9 | Paracentral | 4 | -6 | -21 | 57 | 15.1±1.0 | 13.6±0.9 | 0.56±0.01 | 0.52±0.01 | 1.47±0.02 | 1.45±0.02 | 1.61±0.06 | 1.59±0.05 |
| 10 | Inferior Parietal | 2 | 42 | -39 | 48 | 14.5±1.0 | 13.2±0.7 | 0.53±0.01 | 0.51±0.01 | 1.41±0.01** | 1.34±0.02 | 1.42±0.03 | 1.41±0.04 |
| 11 | Middle Temporal | 21 | 57 | -36 | -3 | 14.7±0.8 | 12.5±0.7 | 0.50±0.01 | 0.48±0.01 | 1.42±0.02 | 1.35±0.02 | 1.51±0.04 | 1.46±0.04 |
| 12 | Supra Marginal | 2 | -60 | -33 | 33 | 14.5±0.8 | 12.8±0.8 | 0.53±0.01 | 0.49±0.01 | 1.41±0.02 | 1.36±0.02 | 1.49±0.05 | 1.42±0.04 |
| 13 | Inferior Frontal | 44 | 42 | 12 | 30 | 12.8±0.7 | 13.6±1.0 | 0.47±0.01 | 0.47±0.01 | 1.37±0.02 | 1.33±0.02 | 1.52±0.05 | 1.43±0.05 |
| 14 | Cerebellum | Crus I | -30 | -72 | -33 | 15.0±1.2 | 12.3±0.7 | 0.52±0.01 | 0.49±0.01 | 1.53±0.02 | 1.45±0.02 | 1.68±0.05 | 1.69±0.05 |
| 15 | Superior Occipital | 19 | -21 | -84 | 24 | 13.8±0.6 | 13.1±0.8 | 0.58±0.01 | 0.54±0.01 | 1.42±0.01 | 1.43±0.02 | 1.57±0.05 | 1.57±0.05 |
| 16 | Middle Occipital | 19 | -33 | -90 | 0 | 13.1±0.7 | 11.9±0.7 | 0.51±0.01 | 0.48±0.01 | 1.25±0.01 | 1.22±0.02 | 1.28±0.03 | 1.26±0.03 |
| 17 | Cerebellum | Crus I | 27 | -69 | -33 | 14.7±1.0 | 11.6±0.7 | 0.52±0.01 | 0.48±0.01 | 1.52±0.02 | 1.45±0.02 | 1.72±0.06 | 1.70±0.05 |
| 18 | Precentral | 4 | -54 | -6 | 33 | 12.9±0.6 | 11.5±0.6 | 0.52±0.01 | 0.50±0.01 | 1.35±0.02 | 1.31±0.02 | 1.45±0.03 | 1.51±0.04 |
| 19 | Supra Marginal | 40 | 57 | -39 | 33 | 12.4±0.6 | 11.9±0.6 | 0.51±0.01 | 0.50±0.01 | 1.38±0.02 | 1.35±0.02 | 1.51±0.04 | 1.41±0.03 |
| 20 | Inferior Frontal | 45 | -48 | 15 | 30 | 12.3±0.6 | 11.8±0.5 | 0.51±0.01 | 0.51±0.01 | 1.42±0.02 | 1.38±0.02 | 1.60±0.04 | 1.48±0.04 |
| 21 | Inferior Frontal | 45 | -42 | 21 | 6 | 13.4±0.8 | 11.3±0.6 | 0.53±0.01 | 0.50±0.01 | 1.50±0.02 | 1.43±0.02 | 1.79±0.05 | 1.83±0.06 |
| 22 | Middle Temporal | 37 | 45 | -69 | 3 | 12.2±0.6 | 10.5±0.7 | 0.49±0.01* | 0.44±0.01 | 1.30±0.01 | 1.24±0.02 | 1.42±0.04 | 1.35±0.04 |
| 23 | Postcentral | 3 | 51 | -12 | 36 | 12.3±0.7 | 10.3±0.5 | 0.47±0.01 | 0.44±0.01 | 1.28±0.02 | 1.22±0.02 | 1.44±0.04 | 1.45±0.04 |
| 24 | Precentral | 4 | -39 | -21 | 57 | 13.1±0.8 | 10.4±0.6 | 0.53±0.02 | 0.48±0.01 | 1.36±0.01 | 1.30±0.02 | 1.54±0.05 | 1.52±0.05 |
| 25 | Postcentral | 3 | -27 | -36 | 63 | 12.4±0.6 | 11.1±0.6 | 0.53±0.01 | 0.50±0.01 | 1.42±0.01* | 1.34±0.02 | 1.54±0.03 | 1.54±0.07 |
| 26 | Lingual | 18 | 18 | -78 | -6 | 11.2±0.5 | 11.7±0.7 | 0.52±0.01 | 0.50±0.02 | 1.37±0.01 | 1.37±0.02 | 1.62±0.05 | 1.58±0.05 |
| 27 | Middle Temporal | 21 | -60 | -30 | -3 | 11.3±0.6 | 10.6±0.6 | 0.48±0.01 | 0.48±0.01 | 1.37±0.02 | 1.32±0.02 | 1.55±0.04 | 1.47±0.04 |
| 28 | Inferior Frontal | 45 | -42 | 45 | 3 | 11.5±0.6 | 9.5±0.4 | 0.47±0.01 | 0.43±0.01 | 1.31±0.01* | 1.24±0.02 | 1.48±0.04 | 1.49±0.04 |
| 29 | Inferior Frontal | 45 | -42 | 42 | 12 | 11.3±0.7 | 9.7±0.4 | 0.47±0.01 | 0.44±0.01 | 1.32±0.01* | 1.26±0.02 | 1.58±0.05 | 1.58±0.06 |
| 30 | Hippocampus | 37 | -27 | -33 | 0 | 11.2±0.6 | 9.4±0.5 | 0.46±0.02 | 0.43±0.01 | 1.33±0.02 | 1.29±0.02 | 1.69±0.07 | 1.59±0.05 |
| 31 | Inferior Frontal | 45 | -45 | 33 | 18 | 10.9±0.5 | 9.7±0.5 | 0.46±0.01 | 0.43±0.01 | 1.34±0.01** | 1.26±0.02 | 1.67±0.06 | 1.57±0.05 |
| 32 | Putamen |  | -24 | 12 | -3 | 11.4±0.6* | 9.1±0.5 | 0.55±0.01** | 0.47±0.02 | 1.49±0.02** | 1.39±0.02 | 1.90±0.06 | 1.91±0.07 |
| 33 | Ant Cingulum | 32 | -3 | 48 | 15 | 11.1±0.6 | 9.7±0.5 | 0.49±0.01 | 0.46±0.01 | 1.45±0.01** | 1.36±0.03 | 1.77±0.05 | 1.66±0.05 |
| 34 | Putamen |  | 18 | 12 | 3 | 11.2±0.8 | 8.8±0.5 | 0.47±0.02* | 0.41±0.01 | 1.39±0.02** | 1.28±0.02 | 1.85±0.07 | 1.80±0.07 |
| 35 | Thalamus |  | -12 | -12 | 6 | 12.3±0.9** | 7.1±0.4 | 0.52±0.02** | 0.36±0.01 | 1.36±0.02** | 1.19±0.02 | 1.64±0.05 | 1.72±0.07 |
| 36 | Middle Temporal | 37 | -48 | -69 | 9 | 11.1±0.6* | 8.8±0.4 | 0.51±0.01** | 0.45±0.01 | 1.34±0.02 | 1.29±0.02 | 1.55±0.04 | 1.55±0.05 |
| 37 | Postcentral | 2 | 21 | -48 | 63 | 10.0±0.4 | 9.8±0.4 | 0.50±0.01 | 0.48±0.01 | 1.37±0.01 | 1.30±0.02 | 1.68±0.06 | 1.47±0.04 |
| 38 | Pons |  | -3 | -30 | -39 | 10.5±0.9 | 8.6±0.7 | 0.36±0.02 | 0.34±0.01 | 1.21±0.03 | 1.22±0.02 | 1.62±0.06 | 1.82±0.11 |
| 39 | Thalamus |  | 12 | -12 | 6 | 12.0±0.9** | 6.9±0.4 | 0.50±0.02** | 0.34±0.01 | 1.35±0.02** | 1.16±0.02 | 1.72±0.05 | 1.70±0.08 |
| 40 | Middle Frontal | 45 | 42 | 42 | 3 | 10.1±0.5* | 8.1±0.4 | 0.44±0.01 | 0.39±0.01 | 1.24±0.02* | 1.15±0.02 | 1.45±0.05 | 1.39±0.05 |
| 41 | Superior Parietal | 7 | 18 | -66 | 51 | 9.4±0.3 | 9.7±0.4 | 0.53±0.01 | 0.52±0.01 | 1.35±0.01 | 1.31±0.02 | 1.54±0.03 | 1.49±0.04 |
| 42 | Middle Frontal | 8 | -27 | 24 | 48 | 10.2±0.5* | 8.8±0.5 | 0.48±0.01 | 0.44±0.01 | 1.38±0.02 | 1.30±0.02 | 1.67±0.04 | 1.70±0.06 |
| 43 | Insula | 13 | 39 | 9 | 6 | 9.7±0.5 | 8.8±0.4 | 0.47±0.01 | 0.44±0.01 | 1.39±0.02 | 1.33±0.02 | 1.87±0.06 | 1.74±0.05 |
| 44 | Hippocampus |  | 24 | -33 | 3 | 9.4±0.4* | 7.9±0.3 | 0.47±0.02 | 0.42±0.01 | 1.30±0.01* | 1.23±0.02 | 1.60±0.04 | 1.62±0.05 |
| 45 | Sup Med Frontal | 9 | -3 | 39 | 45 | 9.3±0.4 | 8.4±0.4 | 0.48±0.01 | 0.46±0.01 | 1.38±0.01* | 1.30±0.02 | 1.86±0.05* | 1.62±0.06 |

*0.001<P ≤ 0.003; **P≤0.001.
