# Supplementary material for: Mapping Small-World Properties through Development in the Human Brain: Disruption in Schizophrenia
Source: PLoS One. 2014 Apr 30;9(4):e96176. doi: 10.1371/journal.pone.0096176 (PMC4005771; doi:10.1371/journal.pone.0096176)
Supplement: Table S3 — Average values in anatomical ROIs for local clustering (lC), characteristic path length (lL), small-worldness (lS) and local degree (lFCD) for healthy adult (CON) and schizophrenia patients (SCZ). (DOCX) [file pone.0096176.s007.docx]

| Regions/BA | *l*C | | *l*L | | *l*S | | *l*FCD | | |
| --- | --- | --- | --- | --- | --- | --- | --- | --- | --- |
|  | **CON** | **SCZ** | **CON** | **SCZ** | **CON** | **SCZ** | **CON** | | **SCZ** |
| Cortical | | | | | | | | | |
| 1 | 0.08 ± 0.01 | 0.07 ± 0.01 | 0.21 ± 0.01 | 0.19 ± 0.01 | 0.24 ± 0.01 | 0.22 ± 0.02 | 1.5 ± 0.1 | | 1.2 ± 0.1 |
| 2 | 0.37 ± 0.01 | 0.34 ± 0.01 | 0.97 ± 0.02 | 0.92 ± 0.02 | 1.08 ± 0.04 | 1.02 ± 0.05 | 8.2 ± 0.5 | | 7.3 ± 0.4 |
| 3 | 0.35 ± 0.01 | 0.31 ± 0.01 | 0.93 ± 0.01 | 0.88 ± 0.02 | 1.03 ± 0.04 | 1.02 ± 0.04 | 8.1 ± 0.5 | | 6.7 ± 0.4 |
| 4 | 0.31 ± 0.01 | 0.28 ± 0.01 | 0.84 ± 0.02 | 0.80 ± 0.02 | 0.97 ± 0.04 | 0.94 ± 0.04 | 7.2 ± 0.4 | | 6.0 ± 0.4 |
| 5 | 0.27 ± 0.01 | 0.25 ± 0.01 | 0.72 ± 0.02 | 0.70 ± 0.02 | 0.86 ± 0.04 | 0.81 ± 0.04 | 5.0 ± 0.2 | | 4.9 ± 0.3 |
| 6 | 0.33 ± 0.01 | 0.30 ± 0.01 | 0.94 ± 0.02 | 0.88 ± 0.02 | 1.13 ± 0.05 | 1.10 ± 0.06 | 7.4 ± 0.4 | | 6.4 ± 0.4 |
| 7 | 0.27 ± 0.01 | 0.26 ± 0.01 | 0.67 ± 0.01 | 0.66 ± 0.02 | 0.75 ± 0.03 | 0.71 ± 0.03 | 5.0 ± 0.2 | | 4.9 ± 0.2 |
| 8 | 0.30 ± 0.01 | 0.27 ± 0.01 | 0.87 ± 0.01 | 0.79 ± 0.02 | 1.09 ± 0.04 | 1.03 ± 0.05 | 6.0 ± 0.3 | | 5.2 ± 0.3 |
| 9 | 0.27 ± 0.01 | 0.25 ± 0.01 | 0.81 ± 0.01 | 0.74 ± 0.02 | 1.02 ± 0.04 | 0.94 ± 0.05 | 5.6 ± 0.3 | | 4.8 ± 0.3 |
| 10 | 0.34 ± 0.01 | 0.31 ± 0.01 | 1.01 ± 0.02 | 0.92 ± 0.03 | 1.26 ± 0.06 | 1.13 ± 0.06 | 6.8 ± 0.4 | | 6.0 ± 0.4 |
| 11 | 0.22 ± 0.01 | 0.19 ± 0.01 | 0.71 ± 0.03 | 0.63 ± 0.03 | 0.91 ± 0.05 | 0.81 ± 0.05 | 4.2 ± 0.3 | | 3.5 ± 0.2 |
| 13 | 0.31 ± 0.01 | 0.29 ± 0.01 | 0.89 ± 0.01 | 0.86 ± 0.02 | 1.13 ± 0.04 | 1.10 ± 0.05 | 6.5 ± 0.3 | | 5.9 ± 0.3 |
| 17 | 0.41 ± 0.01 | 0.38 ± 0.01 | 0.99 ± 0.02 | 1.01 ± 0.02 | 1.09 ± 0.04 | 1.11 ± 0.04 | 9.9 ± 0.5 | | 9.3 ± 0.6 |
| 18 | 0.41 ± 0.01 | 0.38 ± 0.01 | 1.05 ± 0.02 | 1.04 ± 0.02 | 1.18 ± 0.04 | 1.20 ± 0.05 | 8.8 ± 0.4 | | 8.4 ± 0.5 |
| 19 | 0.35 ± 0.01 | 0.33 ± 0.01 | 0.90 ± 0.02 | 0.89 ± 0.02 | 1.03 ± 0.04 | 1.02 ± 0.04 | 7.1 ± 0.4 | | 6.8 ± 0.4 |
| 20 | 0.24 ± 0.01 | 0.23 ± 0.01 | 0.85 ± 0.02 | 0.78 ± 0.03 | 1.11 ± 0.06 | 1.00 ± 0.06 | 4.9 ± 0.3 | | 4.5 ± 0.3 |
| 21 | 0.33 ± 0.01 | 0.31 ± 0.01 | 1.00 ± 0.02 | 0.95 ± 0.02 | 1.23 ± 0.05 | 1.15 ± 0.06 | 7.0 ± 0.4 | | 6.5 ± 0.3 |
| 22 | 0.34 ± 0.01 | 0.33 ± 0.01 | 1.01 ± 0.02 | 0.97 ± 0.03 | 1.23 ± 0.05 | 1.17 ± 0.05 | 7.3 ± 0.4 | | 6.8 ± 0.4 |
| 23 | 0.56 ± 0.01 | 0.56 ± 0.01 | 1.40 ± 0.01 | 1.41 ± 0.02 | 1.53 ± 0.05 | 1.48 ± 0.05 | 14.8 ± 0.8 | | 15.0 ± 0.9 |
| 24 | 0.46 ± 0.01 | 0.42 ± 0.01 | 1.33 ± 0.02 | 1.28 ± 0.02 | 1.78 ± 0.07 | 1.71 ± 0.07 | 9.1 ± 0.5 | | 8.8 ± 0.5 |
| 25 | 0.23 ± 0.01 | 0.19 ± 0.01 | 0.73 ± 0.02 | 0.62 ± 0.03 | 0.94 ± 0.05 | 0.81 ± 0.05 | 4.3 ± 0.3 | | 3.4 ± 0.2 |
| 26 | 0.53 ± 0.01 | 0.51 ± 0.01 | 1.25 ± 0.02 | 1.23 ± 0.02 | 1.24 ± 0.04 | 1.24 ± 0.04 | 17.1 ± 1.1 | | 16.5 ± 1.1 |
| 27 | 0.32 ± 0.01 | 0.31 ± 0.01 | 0.90 ± 0.02 | 0.89 ± 0.03 | 1.12 ± 0.05 | 1.09 ± 0.05 | 5.7 ± 0.3 | | 5.5 ± 0.3 |
| 28 | 0.13 ± 0.01 | 0.10 ± 0.01 | 0.46 ± 0.03 | 0.40 ± 0.03 | 0.58 ± 0.05 | 0.48 ± 0.04 | 2.4 ± 0.2 | | 2.0 ± 0.1 |
| 29 | 0.33 ± 0.01 | 0.33 ± 0.01 | 0.84 ± 0.02 | 0.84 ± 0.02 | 0.94 ± 0.05 | 0.91 ± 0.04 | 6.9 ± 0.4 | | 7.7 ± 0.6 |
| 30 | 0.47 ± 0.01 | 0.46 ± 0.01 | 1.22 ± 0.02 | 1.21 ± 0.02 | 1.40 ± 0.05 | 1.39 ± 0.06 | 11.2 ± 0.6 | | 11.5 ± 0.8 |
| 32 | 0.42 ± 0.01 | 0.38 ± 0.01 | 1.27 ± 0.02 | 1.18 ± 0.02 | 1.67 ± 0.06 | 1.61 ± 0.07 | 8.5 ± 0.4 | | 7.3 ± 0.3 |
| 34 | 0.39 ± 0.02 | 0.33 ± 0.01 | 1.22 ± 0.02 | 1.10 ± 0.03 | 1.71 ± 0.07 | 1.65 ± 0.09 | 7.2 ± 0.4 | | 5.9 ± 0.3 |
| 35 | 0.26 ± 0.01 | 0.22 ± 0.01 | 0.90 ± 0.03 | 0.82 ± 0.03 | 1.20 ± 0.06 | 1.04 ± 0.06 | 4.9 ± 0.3 | | 4.1 ± 0.2 |
| 36 | 0.15 ± 0.01 | 0.14 ± 0.01 | 0.59 ± 0.02 | 0.54 ± 0.02 | 0.80 ± 0.05 | 0.69 ± 0.05 | 3.0 ± 0.2 | | 2.8 ± 0.2 |
| 37 | 0.31 ± 0.01 | 0.29 ± 0.01 | 0.88 ± 0.02 | 0.85 ± 0.02 | 1.07 ± 0.04 | 1.05 ± 0.05 | 6.2 ± 0.3 | | 5.6 ± 0.3 |
| 38 | 0.25 ± 0.01 | 0.23 ± 0.01 | 0.86 ± 0.03 | 0.79 ± 0.03 | 1.15 ± 0.06 | 1.04 ± 0.06 | 4.6 ± 0.2 | | 4.2 ± 0.2 |
| 39 | 0.38 ± 0.01 | 0.36 ± 0.01 | 0.93 ± 0.01 | 0.89 ± 0.02 | 0.97 ± 0.03 | 0.95 ± 0.04 | 8.4 ± 0.4 | | 7.8 ± 0.4 |
| 40 | 0.39 ± 0.01 | 0.37 ± 0.01 | 0.99 ± 0.01 | 0.94 ± 0.02 | 1.04 ± 0.04 | 0.99 ± 0.04 | 8.8 ± 0.5 | | 8.1 ± 0.4 |
| 41 | 0.24 ± 0.01 | 0.23 ± 0.01 | 0.67 ± 0.01 | 0.65 ± 0.01 | 0.77 ± 0.03 | 0.76 ± 0.03 | 5.1 ± 0.3 | | 4.9 ± 0.2 |
| 42 | 0.44 ± 0.01 | 0.44 ± 0.01 | 1.28 ± 0.02 | 1.25 ± 0.02 | 1.49 ± 0.05 | 1.43 ± 0.05 | 10.1 ± 0.5 | | 9.8 ± 0.5 |
| 43 | 0.32 ± 0.01 | 0.28 ± 0.01 | 0.88 ± 0.02 | 0.82 ± 0.02 | 1.01 ± 0.04 | 0.96 ± 0.05 | 7.5 ± 0.4 | | 6.1 ± 0.4 |
| 44 | 0.36 ± 0.01 | 0.35 ± 0.01 | 1.06 ± 0.02 | 1.01 ± 0.02 | 1.25 ± 0.05 | 1.20 ± 0.05 | 7.9 ± 0.4 | | 7.4 ± 0.4 |
| 45 | 0.35 ± 0.01 | 0.33 ± 0.01 | 1.04 ± 0.02 | 0.99 ± 0.02 | 1.29 ± 0.05 | 1.22 ± 0.06 | 7.9 ± 0.4 | | 7.2 ± 0.4 |
| 46 | 0.33 ± 0.01 | 0.31 ± 0.01 | 1.01 ± 0.02 | 0.94 ± 0.02 | 1.28 ± 0.05 | 1.22 ± 0.06 | 7.0 ± 0.3 | | 6.1 ± 0.3 |
| 47 | 0.33 ± 0.01 | 0.30 ± 0.01 | 1.02 ± 0.02 | 0.95 ± 0.02 | 1.31 ± 0.05 | 1.26 ± 0.06 | 7.0 ± 0.4 | | 6.0 ± 0.3 |
| Subcortical | | | | | | | | | |
| Thalamus | 0.39 ± 0.01 | 0.32 ± 0.01 | 1.03 ± 0.02 | 0.94 ± 0.03 | 1.25 ± 0.05 | 1.23 ± 0.06 | | 8.5 ± 0.5 | 6.6 ± 0.4 |
| Caudate | 0.17 ± 0.01 | 0.15 ± 0.01 | 0.50 ± 0.01 | 0.46 ± 0.01 | 0.63 ± 0.03 | 0.58 ± 0.03 | | 3.3 ± 0.2 | 2.8 ± 0.2 |
| Putamen | 0.39 ± 0.01 | 0.34 ± 0.01 | 1.14 ± 0.02 | 1.07 ± 0.02 | 1.60 ± 0.07 | 1.56 ± 0.07 | | 7.9 ± 0.5 | 6.4 ± 0.3 |
| Globus Pallidus | 0.42 ± 0.02 | 0.33 ± 0.02 | 1.27 ± 0.02 | 1.13 ± 0.03 | 1.79 ± 0.08 | 1.67 ± 0.08 | | 8.6 ± 0.6 | 6.4 ± 0.3 |
| Hippocampus | 0.34 ± 0.01 | 0.30 ± 0.01 | 1.05 ± 0.02 | 0.98 ± 0.02 | 1.39 ± 0.06 | 1.31 ± 0.06 | | 6.7 ± 0.3 | 5.8 ± 0.3 |
| ParaHippocampus | 0.31 ± 0.01 | 0.29 ± 0.01 | 1.02 ± 0.03 | 0.96 ± 0.03 | 1.38 ± 0.07 | 1.29 ± 0.07 | | 5.8 ± 0.3 | 5.4 ± 0.3 |
| Amygdala | 0.37 ± 0.01 | 0.31 ± 0.01 | 1.17 ± 0.02 | 1.07 ± 0.03 | 1.64 ± 0.07 | 1.55 ± 0.08 | | 6.6 ± 0.3 | 5.6 ± 0.2 |
| Crus I | 0.32 ± 0.01 | 0.30 ± 0.01 | 0.93 ± 0.03 | 0.88 ± 0.03 | 1.10 ± 0.06 | 1.05 ± 0.06 | | 7.5 ± 0.5 | 6.4 ± 0.4 |
| Crus II | 0.32 ± 0.01 | 0.29 ± 0.01 | 0.98 ± 0.02 | 0.90 ± 0.03 | 1.17 ± 0.05 | 1.10 ± 0.06 | | 7.4 ± 0.5 | 6.1 ± 0.4 |
| Cerebellum III | 0.37 ± 0.01 | 0.36 ± 0.01 | 1.15 ± 0.02 | 1.12 ± 0.03 | 1.52 ± 0.07 | 1.44 ± 0.07 | | 7.9 ± 0.4 | 7.6 ± 0.4 |
| Cerebellum IV-V | 0.40 ± 0.01 | 0.40 ± 0.01 | 1.23 ± 0.02 | 1.23 ± 0.03 | 1.62 ± 0.06 | 1.61 ± 0.08 | | 9.0 ± 0.5 | 9.0 ± 0.5 |
| Cerebellum VI | 0.41 ± 0.01 | 0.40 ± 0.01 | 1.22 ± 0.02 | 1.21 ± 0.03 | 1.57 ± 0.07 | 1.56 ± 0.07 | | 9.3 ± 0.6 | 8.7 ± 0.5 |
| Cerebellum VIIb | 0.29 ± 0.01 | 0.26 ± 0.01 | 1.00 ± 0.02 | 0.91 ± 0.03 | 1.26 ± 0.06 | 1.20 ± 0.07 | | 6.4 ± 0.4 | 5.2 ± 0.3 |
| Cerebellum VIII | 0.35 ± 0.02 | 0.32 ± 0.01 | 1.23 ± 0.03 | 1.13 ± 0.03 | 1.58 ± 0.07 | 1.52 ± 0.08 | | 8.8 ± 0.6 | 7.0 ± 0.4 |
| Cerebellum IX | 0.37 ± 0.02 | 0.33 ± 0.02 | 1.26 ± 0.03 | 1.17 ± 0.03 | 1.65 ± 0.08 | 1.56 ± 0.07 | | 9.4 ± 0.8 | 7.2 ± 0.5 |
| Cerebellum X | 0.18 ± 0.01 | 0.15 ± 0.01 | 0.66 ± 0.02 | 0.60 ± 0.02 | 0.90 ± 0.05 | 0.82 ± 0.05 | | 4.5 ± 0.4 | 3.3 ± 0.2 |
| Vermis | 0.46 ± 0.02 | 0.44 ± 0.02 | 1.30 ± 0.02 | 1.27 ± 0.03 | 1.57 ± 0.06 | 1.53 ± 0.07 | | 11.6 ± 0.8 | 10.5 ± 0.7 |
